# Peer education for HIV prevention among high-risk groups: a systematic review and meta-analysis

**DOI:** 10.1186/s12879-020-05003-9

**Published:** 2020-05-12

**Authors:** Jiayu He, Ying Wang, Zhicheng Du, Jing Liao, Na He, Yuantao Hao

**Affiliations:** 1 Department of Medical Statistics and Epidemiology, School of Public Health, Sun Yat-sen University, Guangzhou, 510080 Guangdong Province China; 2 Department of Epidemiology, School of Public Health, Fudan University, Shanghai, 200032 China; 3 The Key Laboratory of Public Health Safety of Ministry of Education, Fudan University, Shanghai, 200032 China

**Keywords:** Peer education, High risk HIV groups, Meta-analysis

## Abstract

**Background:**

Peer education has become a strategy for health promotion among high-risk groups for HIV infection worldwide. However, the extent to which peer education could have an impact on HIV prevention or the long-term effect of this impact is still unknown. This study thus quantifies the impact of peer education over time among high-risk HIV groups globally.

**Method:**

Following the PRISMA guidelines, a systematic review and meta-analysis was used to assess the effects and duration of peer education. A thorough literature search of PubMed, Web of Science, Embase and Cochrane Library was performed, and studies about peer education on high-risk HIV groups were reviewed. Pooled effects were calculated and the sources of heterogeneity were explored using meta-regression and subgroup analysis.

**Results:**

A total of 60 articles with 96,484 subjects were identified, and peer education was associated with 36% decreased rates of HIV infection among overall high risk groups (OR: 0.64; 95%CI: 0.47–0.87). Peer education can promote HIV testing (OR = 3.19; 95%CI:2.13,4.79) and condom use (OR = 2.66, 95% CI: 2.11–3.36) while reduce equipment sharing (OR = 0.50; 95%CI:0.33,0.75) and unprotected sex (OR = 0.82; 95%CI: 0.72–0.94). Time trend analysis revealed that peer education had a consistent effect on behavior change for over 24 months and the different follow-up times were a source of heterogeneity.

**Conclusion:**

Our study shows that peer education is an effective tool with long-term impact for behavior change among high-risk HIV groups worldwide. Low and middle-income countries are encouraged to conduct large-scale peer education.

## Background

Acquired immune deficiency disease (AIDS), caused by the human immunodeficiency virus (HIV) is a severe infectious disease. High risk HIV groups, including men who have sex with men (MSM), people who inject drugs (IDUs) and female sex workers (FSWs) are those who have high-risk behaviors and thus tend to be disproportionately infected by HIV virus [1]. As the 2018 Joint United Nations Program on HIV/AIDS reported, such high risk groups and their sexual partners accounted for 47% of new HIV infections globally [1]. Compared with the general population, MSM have a 27 times higher infection risk of HIV, IDUs 23 times, and FSWs 13 times [2]. These groups also have a series of high-risk behaviors, such as unprotected sex [2], failing to get HIV tests [3] and sharing drug equipment [4]. Effective behavioral intervention strategies are therefore urgently needed for health promotion among them. One of the key approaches is peer education [5–7].

Peer education is a common strategy for preventing HIV and promoting health worldwide [8] and typically involves recruiting members of a specific at-risk group to encourage members to change risky sexual behaviors and maintain healthy sexual behaviors [9]. What distinguishes peer education from mass media programs is that there is more interpersonal interaction in both directions [9]. Peers are much more likely to influence the behavior of fellow group members since they are assumed to be able to gain a level of trust, which allows for more open discussions on sensitive topics [10, 11]. They also have better access to hidden populations who may have limited interaction with traditional health programs [12]. Finally, they are cost effective in comparison with traditional health-care providers [13, 14].

Previous studies have shown that peer education could reduce risk behaviors [15, 16] and promote health [17–21], but some researchers measured the influence of peer-based intervention on conversations about HIV prevention and highlighted the effect declined and the frequency of conversations on the topics decreased [22]. Previous meta-analyses only synthesized the effect of peer education in developing countries in general population [8] and only revealed the effect of peer education on condom use [17] or HIV testing [23] among MSM groups. What’s more, it is also still unclear whether peer education can bring about positive effects and maintain the changes consistently among different high risk HIV groups both in developed and developing countries.

Consequently, an up-to-date and comprehensive systematic review and meta-analysis is urgently needed to evaluate the effect of peer education on different behaviors among high risk groups. To address these issues, we conducted a systematic review and meta-analysis to examine and summarize the effects of peer education on different behaviors, including condom use, HIV testing, unprotected sex, equipment sharing and, HIV incidence both in developed and developing countries among high risk HIV groups. We also conducted a time analysis of peer education to measure its persistent effects over time.

## Methods

### Search strategy

This systematic review was conducted in accordance with the Preferred Reporting Items for Systematic Reviews and Meta-Analyses (PRISMA) [15]. The literature was searched using four electronic databases: PubMed, Web of Science, EMBASE, and the Cochrane Library. The search included all the literature published from January 2000 to April 2019. We used the search terms (“peer education” OR “peer-led intervention” OR “peer counseling” OR “peer approaches”) AND (“HIV” OR “AIDS”) AND (“MSM” OR “homosexual” OR “IDUs” OR “drug users” OR “FSWs” OR “female entertainment workers”). References of retrieved full-text articles and other reviews were screened for additional eligible publications. All publications were exported to a NoteExpress file and the duplicates were deleted.

### Study selection and eligibility criteria

Studies were selected if they met the following inclusion criteria: (1) peer education intervention related; (2) the intervention was conducted in high-risk HIV groups, including MSM, IDUs and FSWs; (3) original RCTs or quasi-experimental intervention studies or post-intervention studies or serial cross-sectional intervention studies with quantitative data; (4) behavioral, psychological or social outcome(s) related to HIV health promotion; and (5) the article was published in a peer-reviewed journal from January 2000 to April 2019, without language restrictions. We defined peers as demographically-similar counterparts of the target population. Studies were excluded if they were: (1) review or qualitative studies; (2) not presenting outcome data after peer education; (3) conference abstracts or brief reports.

The titles and abstracts were independently reviewed by two authors (He JY and Wang Y), and full texts of potentially eligible studies were downloaded and further screened for final inclusion in our study. When there was uncertainty or disagreement between the two authors as to the eligibility of a study, another author (Hao YT) was invited for guidance in reaching a consensus.

### Data extraction

All data were extracted independently by two authors (He JY and Wang Y) using common abstraction forms. The characteristics recorded for each eligible study included the first author’s name, publication year, study country, study object, mean age of participants, study design, sample sizes, description of intervention in study aims and comparison aims, duration of follow-up, study outcomes, number of events in trial and control groups, outcome indicators, and 95% confidence intervals (CIs).

We then performed a quality assessment of each study included using an 8-point scale which was first used in a meta-analysis in 200 9[8] and has subsequently been cited by other researchers [24–27]. One point was awarded for each of the following items: (1) prospective cohort; (2) control or comparison group; (3) pre/post intervention data; (4) random assignment of participants to the intervention; (5) follow-up rate of 80% or more; (6) comparison groups equivalent in terms of social-demographic measures; (7) comparison groups equivalent at baseline in terms of outcome measures; and (8) sample size≥100. Therefore, the total rigor score for each study ranged from 0 to 8.

### Statistical analysis

Meta-analysis was performed using the package ‘meta’ in R software (version 3.4.3). We converted effect size estimates to the common metric of an odds ratio (OR). ORs and their 95% CIs were extracted directly from reports when available, with adjusted ORs extracted preferentially over unadjusted ORs. If an included study did not report ORs, crude ORs were calculated from extracted data.

The *I*^*2*^ statistic was used to assess the level of heterogeneity across included studies, with values of 50, and 75% representing low to moderate, and high heterogeneity, respectively [16]. Both a fixed-effect model for low heterogeneity studies and a random-effect model for moderate and high heterogeneity studies were used to calculate pooled effect sizes. We explored sources of heterogeneity by performing subgroup analyses among different high risk groups. If substantial heterogeneity was detected (*I*^*2*^ > 90), we performed multivariate meta-regression analyses to investigate the proportion of study variance accounted for by country, follow-up time, and high risk groups. Publication bias was assessed using funnel plots and Egger’s test [28]. We also performed sensitivity analyses by removing one study at a time and recalculating the pooled estimates.

In order to evaluate the time effect of intervention, we developed subgroup analyses of different follow-up times. Different studies reported outcome effects at different follow-up times. Therefore, we conducted meta-analyses of different duration for each intervention result based on the principle of making full use of the information: (1) unprotected sex behavior: 3, 6 and 12 months, (2) equipment sharing behavior: 3, 6, 12, and 24 months, (3) HIV testing behavior: 3, 6, 12, and 24 months, (4) condom use behavior: 3, 6, 12, 24, 36, 48 months, (5) HIV infection: 12, 24, and 48 months. Combined ORs of outcome were calculated in each group. Line charts of combined ORs on each intervention result were conducted to see whether the effect of peer education appeared to decline over time.

### Selection of study endpoints

A meta-analysis was conducted on four behavioral outcomes and one biological outcome which were reported across multiple studies: HIV testing, condom use, injection drug equipment sharing, unprotected sex, and HIV measure. HIV testing was the dichotomous proportion of respondents who did or did not have an HIV test. Condom use was always measured as a multiple categorical variable in the studies, such as used condom in last sexual encounter, always used condom, used condom with clients, used condom with sexual partners etc. We prioritized the general and comprehensive variable which can most reduce risk and represent the most cases, such as always used condom. We also conducted subgroup analyses on condom use including condom use with regular partners, condom use with casual partners and consistent condom use. Intravenous drug equipment sharing included reported episodes of sharing needles/syringes, rinse water, and/or cooking utensils. HIV measures including incidence and prevalence, were measured by self-reports, chart reviews, and clinical diagnoses. For all outcomes, our selection of the outcomes to be included in the meta-analysis prioritized the comparison with the longest follow-up time.

## Results

### Search and description of studies

**Fig. 1:**
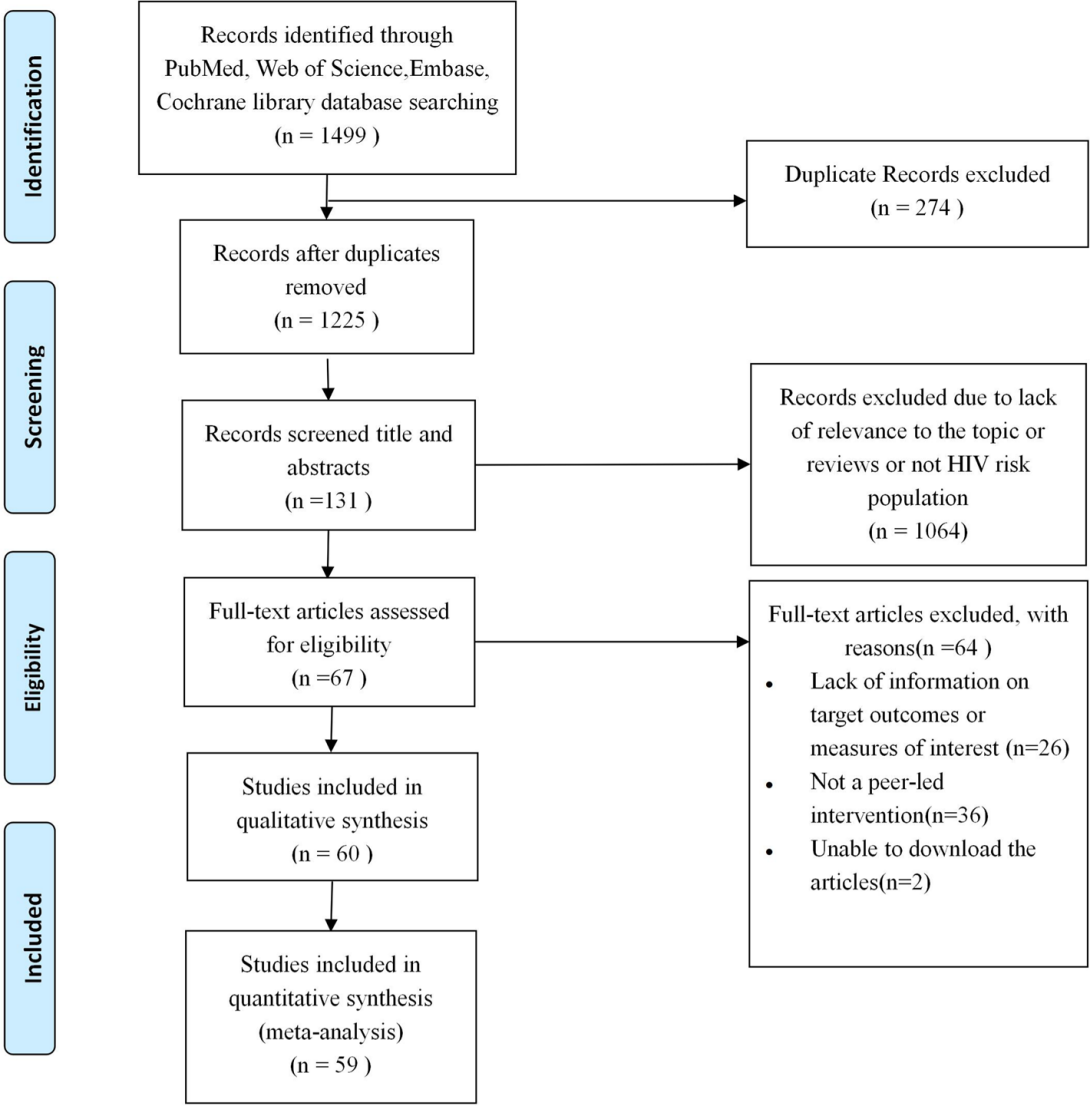
Flow chart of literature search and selection of studies

The initial search of our chosen four electronic databases yielded 1499 articles; of which 274 duplicates were removed. Of the remaining 1225, 1064 were excluded due to lack of relevance to peer education, the target population i.e. not HIV risk groups, or because they were reviews. Full text screening of the remaining 131 papers led to the further exclusion of 64 papers for the following reasons: lack of information on target outcomes (*n* = 26), not a peer-led intervention (*n* = 36), and two articles could not be downloaded. Thus, 60 studies met our predefined inclusion criteria (Fig. 1). One of these 60 studies was not included in the meta-analysis because it lacked quantitative data. The characteristics of each study are detailed in Table 1. The information of the selected studies can be found at Supplemental material 1.

**Table 1:** Study description

Study	Country & Groups	Study design	Intervention description	Comparison	Follow-uptime
M. A. Davey-Rothwell 2011	the US, FSWs	RCT	The social network based intervention defined a Peer Mentor as someone who had meaningful conversations with partners, friends, and other people in their social networks. It consisted of five small group sessions and one individual session. Each group session included facilitated discussions, presentation of new information, and Peer Mentoring practice activities like role-plays.	One group session delivered by a female facilitator. The session, lasting about 90 minutes, focused on HIV and sexually-transmitted infections (STIs) transmission and risk reduction information and consisted of didactic presentations and a culminating game.	6,12,18 months
Hilary L. Surratt 2014	the US, FSWs	RCT	A strengths-Based / Professional-Peer (PP) Condition. The peer facilitator participated in the intervention sessions and remained in contact with their cases throughout the entire six months study participation period, providing ongoing support for service linkage.	A strengths-based / professional-only (PO) condition, in which a professional case manager partnered with the participant.	3,6 months
Scott D. Rhodes 2011	the US, heterosexua lly active people	RCT	The HIV intervention was peer-led; three people from rural areas were recruited and trained to serve as peer educators. They were trained in four sessions including the epidemiology of HIV and health disparities, HIV transmission, risk behavior, cultural and social influences on sexual health, access to health-care services, predictors of behavior change, and group facilitation.	The cancer education comparison intervention was delivered in one 2-hour session and focused on prevention of cancers particularly relevant to men: prostate, lung, and colorectal cancers.	3,12 months
Theresa H Hoke 2007	Madagascar, sex workers	RCT	Peer educators trained by the study provided all participants risk reduction counselling that included condom promotion. Peer educators were in frequent contact with supervising clinic personnel. The study manager also met monthly with peer educators to monitor and encourage mastery of counselling and other duties.	Peer& clinic arm received bi-monthly clinic counselling sessions, delivered by a nurse and a physician and led by a consultant specializing in behaviour change contact and lasting about 15 minutes.	12 months
Ishika Basu 2004	India, sex workers	quasi-R CT	Intervention: Standardizing resources and the enhanced intervention groups included training by a team of local peer educators. Goals were to build skills and confidence in providing education and foster empowerment for local sexual workers.	Only standardizing resources, including health clinics and health care services	6,12,16 months
Alfonso Ang 2012	Philippines,FSWs	RCT	The first group was the peer education intervention group which received a special intervention consisting of peer leader training, role playing for development of skills, etc. The second group consisted of managers of the officers. The third intervention group received both peer education and managerial training intervention.	The control group received usual educational materials and presentations by the social hygiene clinic nurses.	12 months
Vivian F.Go 2013	Vietnam, IDUs	RCT	The experimental intervention consisted of six 2-h, small group, peer educator training sessions during a six-week period and three booster sessions. The intervention is modeled on Self-Help in Eliminating Life-threatening Diseases, a peer network-oriented intervention that successfully reduced injecting and sexual behaviors.	Control: standard of care and government sponsored pamphlets on HIV.	3,6,12 months
Dianmin Kang 2013	China, FSWs	Post-intervention cross-sectionalsurvey	The integrated individual level intervention, community mobilization and structural interventions included 100% condom promotion activities, outreach and peer education to promote risk and health-seeking behavior change, to expand HIV testing and promote standardization of clinical STI services, monitoring and encouraging adherence.	Control: the standard of care, the routine testing, prevention, but not the integrated individual, community, and structural intervention.	5 years
Xiushi Yang 2011	China, FSWs	A cluster RCT	The intervention condition consisted of small group, peer-assisted multiple sessions that aimed to improve HIV knowledge, enhance personal motivation to take preventive actions, develop preventive behavioral skills. The interventions were five 60–90-minute multimedia group sessions, which were broken down to multiple mini sessions of 45–50 minutes each and delivered, once a week, on-site in the establishment.	The control condition was a group-based didactic education. The informational education was divided into the same number of mini sessions which were conducted by trained health educators.	3 months
Carl Latkin 2009	Thailand,IDUs	RCT	Six two-hour, small-group, network-oriented peer educator training sessions during a four week period and two booster sessions at six and 12 months. The sessions included instruction in methods of harm reduction, developing and practicing communication skills and strategies, role-plays, and problem solving exercises. At each session, participants developed a plan about how they would discuss and encourage injection and sexual risk reduction with the specific network members.	Control: no intervention beyond volunteer counselling testing was received	6 months
D.W.Purcell 2007	the US, IDUs	RCT	A 10-session peer mentoring intervention	An 8-session video discussion intervention.	3,6,12 months
M.Yun Gao 2007	China, MSM	A quasi-experiment	Gave the intervention program with conducting peer-led participatory activities, and tested again. The stories in the series represented key issues on the exposure to epidemics in the gay community and strategically promoted awareness of STIs and HIV prevention. Three small media materials provided further support for relevant educational messages. Based on gay men interests, outdoor edutainment activities were organized to expand their networks and get them to make friends.	Received no intervention information, and were tested again .	6 months
Nai-Ying Ko 2013	China, Taiwan, MSM	A quasi-experiment	The 369 internet Popular Opinion Leaders (iPOLs) were trained by HIV/STI experts during a 12-week period. The iPOLs platform used 2-way communication format on Facebook in which the iPOLs shared and exchanged news, video clips, reports, and opinions, and had the capability to connect with others for advice and support. Intervention: 2-way conversations related to risky behaviors on the online iPOLs platform were reviewed by iPOLs, discussed, and reinforced at subsequent iPOLs training sessions.	Control: no intervention	6 months
Sean D. Young 2013	the US, MSM	A cluster RCT	Intervention: sixteen peer leaders were randomly assigned to deliver information about HIV or general health to participants via Facebook groups over 12 weeks. Peers delivered information on HIV prevention.	Peer delivered information on general health over Facebook.	12 weeks
Sean D. Young 2015	Peru, MSM	A cluster RCT	Intervention: peer leaders would attempt to interact with participants about the importance of HIV prevention and testing.	Enhanced standard of care provided by local community clinics and government.	12 weeks
Hongjing Yan 2014	China, MSM	Cohort study	An MSM peer-led, community-based organization (CBO), mobile rapid HIV testing program. MSM peers were trained and certified in HIV counselling for pretest and post-test sessions. After discussing the means of acquiring and preventing HIV infection and the clients’ behaviour, individualized risk reduction counselling was done. Following pretest counselling, a finger-prick rapid HIV test was performed by the peer in the private room or van and screening test results were disclosed with post-test counselling.	Comparison: surveillance surveys	6 months
Marco A. Hidalgo 2015	the US, MSM	RCT	Intervention: Male Youth Pursuing Empowerment, Education and Prevention around Sexuality (MyPEEPS) is a group-level intervention consisting of 6 modular, interactive, group sessions (2 h each) and delivering twice weekly for 3 weeks. Two gay / bisexual male facilitators, both of whom had extensive experience leading group-based interventions among lesbian, gay, bisexual, and transgender (LGBT) youth.	A group level intervention focused on HIV risk reduction but relied entirely on a lecture format led by facilitator.	6,12 weeks
Joseph T.F Lau 2013	HongKong, China, MSM	A cluster RCT	Intervention: The seed peer educator (SPE) delivered manual-based and theory-based peer education to their fellow social network members (SNM) during the 2-month study period. The trained SPE delivered the theme-based HIV prevention messages to their SNM via daily social contacts, using the standard scripts as references. Samples of phone text messages and emails were provided to the SPE but flexibility was allowed. Repetitions in different wordings and settings were encouraged.	Control: SPE gave some printed education materials to their fellow SNM.	6 months
Guodong Mi 2015	China, Chengdu, MSM	A quasi-RCT	Four complementary modules: an information exchange website, a bulletin board program, individualized one-on-one online counseling, and an animated game. The individualized one-on-one online counseling was conducted by eight peer educators through Tencent QQ every evening during the study period. Peer educators summarized and posted answers to frequently asked questions on the information exchange website.	Control: followed standard-of care procedures and did not receive an intervention.	6 months
Carl Latkin 2013	the US, IDUs	RCT	Six 2-hour peer-educator sessions and two booster sessions. The training focused on teaching indexes 1) how to promote safer sex and drug injection skills among network members, and 2) promote norms about HIV risk reduction with their members. Participants engaged in role-plays to practice their risk reduction skills. Participants were encouraged to model safer behaviors when they were with their peers. A major component of the training focused on developing communication skills on how to talk with network members about risk reduction.	Participants randomized into the control condition did not participate in any intervention sessions.	24 months
Richard S. Garfein 2007	the US, IDUs	RCT	The intervention consisted of six 2-h sessions. Session 1 described HIV and HCV transmission through sex and injection drug use, informed participants about disease prevalence in their communities, and described the vital role peer educators play in preventing further disease spread. Sessions 2 and 3 provided peer education about safer injection and sexual practices. Session 4 added skills-building activities. During session 5, small teams of up to five participants conducted 90-min peer education sessions. Session 6 consisted of a large group debriefing, goal-setting to encourage continued risk reduction.	A video discussion intervention (VDI). Participants watched hour-long films addressing social and health issues followed by facilitated discussion using scripted questions.	6 months
Irving F. Hoffman 2013	Russia, IDUs	RCT	Indexes attended eight training sessions aimed at understanding the risks associated with injecting drug use and unprotected sex, and development of safe behavior skills. After completing the training, index participants from the experimental group were invited to repeat (booster) meetings once a month for 4 months to discuss their strategies, successes and failures in implementing information transfer to their network members.	Eight sessions of equivalent length devoted to discussing issues of interest, viewing lifestyle videos, practicing non-specific exercises. No booster sessions were offered.	2 years
Karin Elizabeth Tobin 2011	the US, IDUs	RCT	The STEP into Action intervention sought to train active injection drug users to be Health Educators and focus to individuals in their personal social network who inject and/or are sexual partners. The intervention condition focused on promoting risk reduction with personal risk network and consisted of 5 group-based, one individual and one session with the Index participant. Content of the group sessions focused on increasing skills to reduce injection communication skills to conduct outreach to personal risk networks.	5 group-based sessions that addressed injection drug-use related topics. The sessions were informational and did not include skills training.	6,12,18 months
Aleksandra Mihailovic 2015	the US, IDUs	RCT	The intervention consisted of information about HIV prevention and teaching participants the skills needed to promote risk reduction within their personal risk networks. It had seven sessions, five of which were group-based. The topics were: introduction to the health educator role and communication, reduction of injection and drug splitting risk behavior, etc. All sessions were imparted by peer educators.	Five-group sessions during which participants received information on injection-drug use topics, but were not taught skills for HIV risk reduction.	6,12,18 months
Mary E. Mackesy-Amiti 2012	the US, IDUs	RCT	In the first sessions, participants learned what it meant to be a peer educator. The first two sessions focused on injection-related risk and the third and fourth sessions focused on sexual risk behavior. In the fifth session, participants were given an opportunity to practice sharing risk-reduction information in a community setting. The sixth session consisted of a group debriefing about the community-based peer education session.	Watching videos followed by facilitated discussion for an equivalent amount of time as the intervention group. Videos addressing social and health issues were chosen to be of interest to the target population.	6 months
Robert E. Booth 2016	Ukraine, IDUs	RCT	Intervention training consisted of five sessions delivered in small groups over a 2 week period designed to motivate peer leaders to become educators within their injection network and provide them with skills training in how to teach HIV risk reduction behaviors to network members effectively. Peer leaders were encouraged to model safe behaviors with their network members. Training sessions consisted of role playing and other interactive learning techniques.	The testing and counselling intervention, Ukraine’s standard of care.	12 months
Carl A Latkin 2003	the US, IDUs	RCT	10-sessions program and each session last 90 min. The training program incorporated cognitive-behavioral intervention components. Everybody study focused on drug risk reduction among risk networks. Role-playing and other safer sex exercises were included to increase participants’ comfort level in discussing and using condoms. Participants reviewed skills and made a public commitment to continue the learning process and peer education.	The initial session contained basic HIV prevention education. Each of the other control sessions consisted of a 1-hr videotaped presentation and 30-min group discussion.	6 months
Song-Ying Shen 2011	China, IDUs	Pilot intervention	The peer-based behavioral intervention was organized by physician, nurse, and coordinator. The intervention program included: recruitment, training peer educators, and implementation: (1) delivering one-on-one training to peers; (2) engaging in exercises focusing on injection and sexual risk behaviors; (3) activities designed to get participants to practice new skills.	Control: routine HIV/STI education consisted of police personnel handing out educational pamphlets and providing lectures focused on HIV/STI education	3 months
Robert E. Booth 2011	Ukraine, IDUs	Quasi-RCT	The intervention consisted of 5 sessions led by outreach workers, delivered in small groups over 2 weeks, designed to empower peer educators to be mentors and provide them with training in how to effectively motivate their network members to reduce HIV risk behaviors. Network members typically accompanied their peer educator to the NGOs in groups. Each highly scripted session included role playing and other interactive learning techniques and exercises.	Individually-based: Injection drug users were asked where they saw them selves on each hierarchy and, together with outreach workers, asked to discuss what they could do to move to lower-risk positions.	2 years
Susan G. Sherman 2009	Thailand, IDUs	RCT	The peer education condition aimed to teach participants to think critically about and reduce their methamphetamine use and sexual risk behaviors. Participants were taught communication skills that they practiced in role plays during the sessions and risk reduction messages to specific social network members that were identified through a social network inventory administered at baseline.	The sessions focused on the causes and consequences of methamphetamine use. The sessions placed no emphasis on discussing the session content with social network members.	3,6,9,12 months
Yu Liu 2018	China, MSM	One-on one RCT	Participants would receive a message sent by the designated peer counsellor social media apps to schedule a mutually confirmed time within the buffering period. The peer-counselling session involved a one-on-one 60-minute discussion focusing on topics regarding specific high-risk behaviors modification, including the strategy to reduce male/female sexual partners, condomless anal/oral sex, commercial sex. At the end of the counselling, peer counsellor and participant would identify one or more goals for safer sex to be qualitatively evaluated in the next visit.	Standard of care (SOC) participants received counselling provided by a CDC trained doctor. A generic message reminder was sent to the participants 3 days prior to the scheduled visit. The 30-min SOC counselling covered contents related to safer sex and prevention of HIV transmission per China CDC’s HIV counselling guidelines.	3 months
Yuwen Duan 2013	China, MSM	Quasi-RCT	A 12-month community level intervention involving MSM popular opinion leaders (POLs) to advocate for safer sexual behaviors to MSM community members and distribute HIV-education materials and condoms in targeted MSM-themed venues. Influential and respected peer leaders were used.	Standard HIV prevention activities in accordance with national and municipal policy including availability of HIV related health education materials and voluntary HIV counseling and testing services at health clinics.	12 months
R.S.Broadhead 2006	Russia, IDUs	A quasi-experiment	In the Standard-PDI, IDU-recruiters are offered nominal monetary rewards for both recruiting peers and educating them in a body of prevention information. (PDI: peer education intervention)	In the Simplified-PDI, IDU-recruiters are asked to educate and recruit their peers, but the reward for recruiting is woven into their education efforts. This modification made it 50% less expensive to operate in respondent fees.	6 months
Study	Country & groups	Study design	Intervention description		Follow-up time
S Thilakavachi 2011	India, FSWs	A serial cross-sectional	study Avahan program, the India AIDS Initiative, a large-scale HIV prevention program, including: (1) peer-mediated outreach to identify and address difficulties reported by FSW, plus behavior change communication to promote condom use and regular STI screening; (2) establishment of dedicated sexual health services for FSW and their regular partners, offering STI identification and syndrome case management, etc.		3 years
B. Ramesh2010 Hari Kumar.2011 Mandar M. 2011	India, FSWs	A serial cross-sectional study	The Avahan program mentioned above.		3 years
P. Goswami 2012 T.Subramanian 2013	India, MSM	A serial cross-sectional	study The Avahan program mentioned above.		4 years
Shajy Isac 2015	India, FSWs	A serial cross-sectional	study The main interventions included peer-led community outreach and community mobilization. Three main strategies were employed to address HIV prevention among FSWs and their clients: promotion of safer sex behavior through a peer-mediated communications strategy; and enhancement of the enabling environment for the adoption of safer sex practices.		8 years
Hongbo Zhang 2010	China, MSM	Cohort study	A peer-driven behavioral intervention was chosen to influence the MSM peer networks. Each intervention group consists of a seed and his referral chain made up of his peers, consisting of 4 1.5-hour sessions with activities such as role playing, games, group discussions, brain storming, and competitions to test knowledge.		3 months
Isidore T Traore 2015	Burkina, FSWs	A prospective, intervention cohort study	The intervention combined prevention and care within the same setting, consisting of peer-led education sessions, psychological support etc. Peer-led education sessions were conducted every day at the study clinic and weekly in the sex work venues, addressing seven themes including HIV testing, STI diagnosis and treatment, genital herpes, condom use.		2 years
Stanley Luchters 2008	Kenya, FSWs	Pre and post intervention cross-sectional surveys	Peer educators conducted one-on-one or weekly-group sessions. Peer-led activities occurred throughout the five-year period at a relatively constant rate. Peer educators also led monthly community gatherings with active participation of FSW, youth and other community members. They provided HIV education, condom promotion and other risk reduction activities and were accompanied by mobile volunteer-based testing services, facilitating entry to HIV testing.		5 years
Xue haoming 2015	Vietnem, FSWs	Serial cross-sectional study	Peer advise, training, lectures, interactive games, free condom distribution were provided		5 years
Simran Shaikh 2016	China, FSWs	a pre- and post-intervention cross-sectional survey design	Interventions included behavior change communication such as outreach-based interpersonal communication through peers, media posters and so on. These communications provide counselling and information on safe sex, STIs, condom use, HIV testing. The program provides three broad categories of activities. The first focuses on improving the organizational and technical capacity of community-based organizations (CBOs) working with transgender communities. The second is to support CBOs to provide a range of basic community-based prevention and linkage to care interventions.		4 years
Zhu Junli 2008	China, Anhui, MSM	cohort study	Intervention based on the combination of initiator-led and peer-driven effects. Stage 1: risk assessment; stage 2: develop safety behavior plan; stage 3: behavior change practice; stage 4: behavior change enhancement		3 months
Duangta Oawa 2013	Thailand, FSWs	A quasi-experimental study	Population Services International (PSI) has implemented the Sisters program to prevent HIV among transgender women in the city of Pattaya. Sisters offers a drop-in center (DiC) that provides counseling, and on-site HIV and STI testing. Sisters also engages in peer-led interpersonal communication. Peer educators meet clients in a safe, private location for counseling, psycho-social/emotional support, and information on gender reassignment, and cosmetic surgery. Peer educators facilitate linkages to transgender-friendly government health services and will accompany.		12 months
Issouf Konate 2011	Burkina Faso, FSWs	Cohort study	Six peer leaders, supervised by a coordinator, were trained to lead group education sessions. Each peer leader supervised 5 field workers peers who carried out more individually tailored information, education and communication sessions at working sites day and night, identified potential participants, and maintained adherence to follow-up visits.		12 months
Scott Geibel 2012	Kenya, sex workers	Two independent cross-sectional surveys	40 peer educators were recruited and trained in HIV prevention. All the peer educators were given additional training in basic counselling skills. The peer educators also attended workshops on alcohol and drug harm reduction related to HIV prevention. Approximately 1900 male sex workers and non-sex worker MSM contacts were recorded by the peer educators. Activities during these contact sessions included brief counselling, health referrals and/or condom/lubricant distribution.		12 months
Yuri A. Amirkhanian 2005	Russia, Bulgaria, MSM	A randomized social network HIV prevention trial	Experimental condition social network leader attended a group training program with five to nine leaders of other networks. The aim was to establish regular HIV prevention communication between the leaders and their network members. Trainers used behavioral techniques to help network leaders gain skill and comfort in delivering HIV prevention messages. Control: no intervention.		3 months
AL Wietz 2015	Malawi, MSM	Cohort study	Intervention: individual, health-care, and community levels. The individual-level component included outreach and education provided by 10 trained peer educators and aimed to reduce behavioral risks for HIV and improve use of HIV prevention methods. The health sector intervention focused on providing an intensive training with pre- and post-test evaluation of 25 staff. The community-level intervention focused on capacity building through the empowerment of peer educators with an aim to increase community permeation of HIV prevention packages.		10,13,16 months
Lisa M Williamson 2001	British, MSM	Cohort study	The Gay Men’s Task Force (GMTF) initiative trained 42 peer educators to work in bars contacting homosexual men. The peer educators wore distinctive uniforms and would distribute GMTF leaflets on sexual health and behavioral issues and then approach men to discuss both these and wider issues along with advocating sexual health service uptake. A contact involved a conversation between a peer educator and a customer in the bar, where issues raised by both would be discussed and further leaflets distributed if required. The resultant discussions covered a wide range of health-related topics and while these did mainly reflect the content of the leaflets, other issues related to sexually transmitted infections and condoms and lubricants were also raised.		9 months
Don C. Des Jarlais 2007	Vietnam China, IDUs	Serial cross-sectional surveys	The intervention follows a peer outreach model developed in the United States. The peer educators regularly contact other IDU in the community and provide them with information on reducing drug use and sexual risk behaviors. They distribute sterile needles and syringes, sterile water for injection, condoms, and no-cost vouchers that can be redeemed for sterile injection equipment and condoms in participating local pharmacies. The peer educators also collect and safely dispose of used needles/syringes directly from drug injectors at injecting sites in the community.		6,12,18,24 months
Sylvia Abebajo 2015	Nigeria, IDUs, MSM	Serial cross-sectional analysis	A standard mobile outreach service, was designed to provide HCT services through a network of mobile community-based key opinion leaders (KOLs). KOLs were male most-at-risk-populations (M-MARPs) community influencers and mobilizers who were trained as peer educators to deliver the minimum prevention package intervention (MPPI) to community members.		3 years
Bindya Jain 2014	India, IDUs	Cohort study	Each peer educator is required to meet five to six IDUs a day during one-to-one or group education sessions. While one-to-one sessions generally last for 15-30 min, group sessions are longer (30–45 min), depending on the topics covered and the type of services provided during the session. Peer educators primarily counsel IDUs on behavior change including safe needle-syringe use and safe sex for the prevention of HIV and other sexually transmitted infections (STI).		2 years
Theodore M.Hammett 2005	China, Northern Vietnam, IDUs	Serial cross-sectional study	The peer educators, who receive initial and refresher training, provide IDUs with HIV risk reduction information and distribute new needles/syringes and condoms and vouchers redeemable in participating pharmacies for new needles/syringes and condoms. The peer educators also collect and dispose of used needles/syringes.		18 months
Theodore M.Hammett 2012	China,Vietnam, IDUs	Serial cross-sectional study	The peer educators regularly contact other IDUs in the community and provide them with information on reducing drug use- and sex-related HIV risks, verbally and through distribution of brochures. They distribute new needles/syringes, ampoule of sterile water for injection solution, and condoms and vouchers redeemable for these items at participating pharmacies. The peer educators also perform a valuable public health service by collecting and disposing of used needles/syringes that might otherwise put community residents at risk. In addition, the peer educators' weekly meetings include training on special topics. The peer educators are supervised locally by health department staffs.		6, 12, 24, 48, 72, 84 months
Theodore M.Hammett 2011	Vietnam, IDUs	Serial cross-sectional study	The project employs a peer district coordinator and 4–5 other peer educators (PE) in each district. Each PE has an active caseload of approximately 50 sexual partners (SPs). The PEs assess the needs and situations of each SP and provide risk reduction information, materials, commodities, and referrals tailored to each client. PEs also promote other HIV prevention approaches including lower-risk sexual activity and ARV treatment for the male partner where indicated, with high adherence. Regular HIV testing is also promoted through referrals to fixed site and mobile VCT.		12, 24 months
Katherine P. Theall 2015	the US, IDUs	Two rounds cross-sectional studies	The study design consisted of two phases. During Phase 1, ethnographic research was conducted and to obtain baseline data on target community members. Phase 2 of the study consisted of the implementation and evaluation of the community-based popular opinion leaders (c-POL) intervention. POL training consisted of two 90-minute group sessions, one week apart, led by both staff depending on the C-POLs-female field staff for female POLs and male field staff for male POLs. In session one the leaders reviewed basic epidemiology of HIV infection, high-risk behaviors and risk reduction techniques, and began training to provide effective health promotion and prevention messages. Session two continued training as well as social skill rehearsals or role-play of techniques learned.		6 months
Margaret R.Weeks 2009	the US, IDUs	Cohort study	Project risk avoidance partnership (RAP) implemented a two-level intervention program. At the first (staff delivered) level, peer health advocates (PHAs) received the RAP Peer Health Advocacy Training Curriculum which was a 10-session, theoretically driven interactive training program. The second-level of RAP intervention was the modular program PHAs delivered to their peers, called the RAP Peer-delivered Intervention. This required PHAs to engage recipients during each interactive encounter in at least two of three primary intervention components: 1) provision of prevention education, 2) demonstration of proper prevention practices, and/or 3) delivery of prevention materials.		6 months

Of these 60 studies, 34 articles employed randomized controlled trials or quasi-experiments, and 9 articles were cohort studies. The remaining 18 studies were serial cross-sectional studies. As shown in Table 1, 25 studies were conducted in East and Southeast Asia, 9 in Central Asia, 15 in North America, eight in Africa, 7 in Europe, and one in South America with some studies conducted across two countries.

Target populations included MSM (*n* = 18) [12, 17–21, 32–43], injected drug users (*n* = 22) [22, 44–64], female sexual workers (*n* = 20) [29–31, 65–80]. The included studies were undertaken between 2001 and 2009, and the population number ranged from 69 to 7015. Mean or median age varied from 16 to 43 years. Study quality assessment scores ranged from 2 to 8, with a mean score of 5.05 out of 8 which is the most rigorous (Supplemental Table 1). According to the sensitivity analysis, the results were robust after moving each study (Supplemental Figure 1, 2, 3, 4, 5, 6, 7, 8) and the Egger tests indicated that there was no publication bias, which are shown in the supplemental material (Supplemental Table 2).

### Impact of peer education on outcome measures

Table 2 presents a summary of the pooled effect sizes for the five outcomes, including overall effects, effects stratified by the three target populations, as well as the level of a country’s economic/social development.

**Table 2:** Summary of meta-analysis by outcome, target population and country development

	HIV testing	Equipment sharing	Unprotected sex	Condom use	Condom use with regular partners	Condom use with casual partners	Consistent condom use	HIV prevalence
Size of population	12775	13830	6289	46130	36622	34589	41582	28061
Overall pooled effect size (95%CI)	3.19* (2.13,4.79)	0.52* (0.35,0.76)	0.82* (0.72,0.94)	2.66* (2.11,3.36)	2.45* (1.64,3.66)	2.79* (2.13,3.66)	1.80* (1.47,2.21)	0.64* (0.47,0.87)
I 2 for heterogeneity	92%	93%	50%	90%	95%	84%	86%	83%
Pooled effect size by target population								
MSM	3.71* (2.09,6.57)	NA	0.51* (0.34,0.76)	1.59* (1.42,1.78)	2.22* (1.54,3.20)	2.21* (1.32,3.71)	1.26* (1.07,1.66)	0.94 (0.58,1.52)
IDUs	2.86* (2.07,3.97)	0.52* (0.35,0.76)	0.92 (0.80,1.07)	2.84* (1.08,7.48)	NA	7.13 (1.97,25.83)	1.03 (0.78,1.35)	0.46* (0.34,0.63)
FSWs	2.76* (1.08,7.07)	NA	0.41* (0.21,0.77)	3.19* (2.41,4.23)	2.40* (1.32,4.35)	3.07 (2.40,3.93)	2.44* (1.85,3.21)	0.75 (0.51,1.09)
Pooled effect size by development								
Developed country	1.59* (1.05,2.42)	0.71* (0.57,0.88)	0.82* (0.70,0.95)	1.87* (1.44,2.41)	NA	NA	NA	NA
Developing country	4.07* (2.68,6.16)	0.31* (0.14,0.71)	0.67 (0.35,1.28)	2.58*(2.01,3.30)	2.45*(1.64,3.66)	2.71*(2.07,3.56)	1.91* (1.48,2.46)	0.67* (0.47,0.96)

*p < 0.05; *NA* not applicable to structure

#### HIV testing

**Fig. 2:**
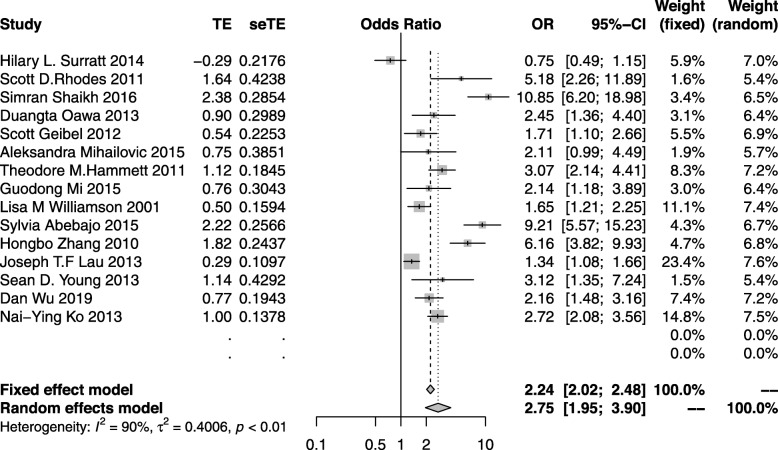
The forest plot of the HIV testing

Fifteen studies [18, 19, 22, 30–32, 34, 35, 39, 43, 51, 76, 79, 80] reported the quantitative outcomes on HIV testing with a combined study population of 12,775 and two studies did not show an increase rate of HIV testing. The outcome of the random effect model suggested that the effect was significant (OR: 3.19; 95%CI: 2.13–4.79) with substantial heterogeneity across studies (*I*^*2*^ = 92%). This thus indicates that the peer education was able to increase the rate of HIV testing among high risk HIV groups globally (Fig. 2).

#### Equipment sharing

**Fig. 3:**
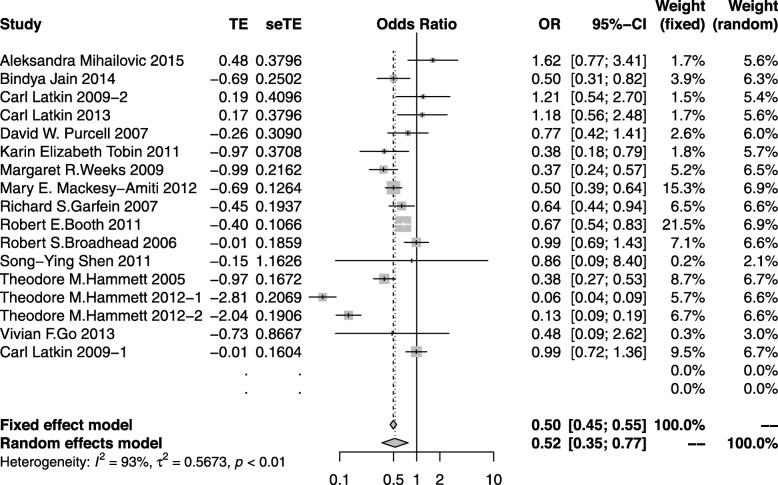
The forest plot of the equipment sharing

Sixteen studies [22, 45–47, 49–55, 57–59, 61, 62] generated 17 discrete effect sizes on equipment sharing with a combined study population of 13,830. Although seven of the sixteen studies reported non-significant changes in equipment sharing before and after intervention, the outcome of the random effect model indicated that the overall effect was significant (OR: 0.52; 95%CI: 0.35–0.76) with substantial heterogeneity across studies (*I*^*2*^ = 93%). The meta-analysis of these 16 articles suggested that through peer education, IDUs would reduce equipment sharing (Fig. 3).

#### Unprotected sex

**Fig. 4:**
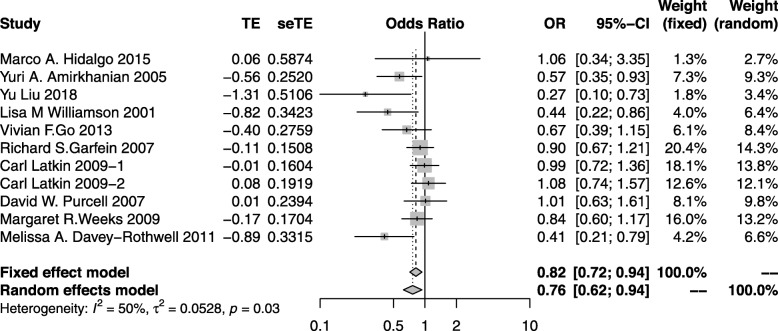
The forest plot of the unprotected sex

Ten studies generated [17, 20, 29, 42, 43, 46, 55, 57–59] 11 independent effect sizes on unprotected sex with a combined study population of 6289. Four of the articles showed a significant reduction in unprotected sex, while four of the articles showed a non-significant reduction. Another three studies found no changes before and after peer education intervention. The fixed effect meta-analysis model showed that peer education lowered 18% of unprotected sex among high risk groups worldwide (OR: 0.82; 95%CI: 0.72–0.94; *I*^*2*^ = 50%) (Fig. 4).

#### Condom use

**Fig. 5:**
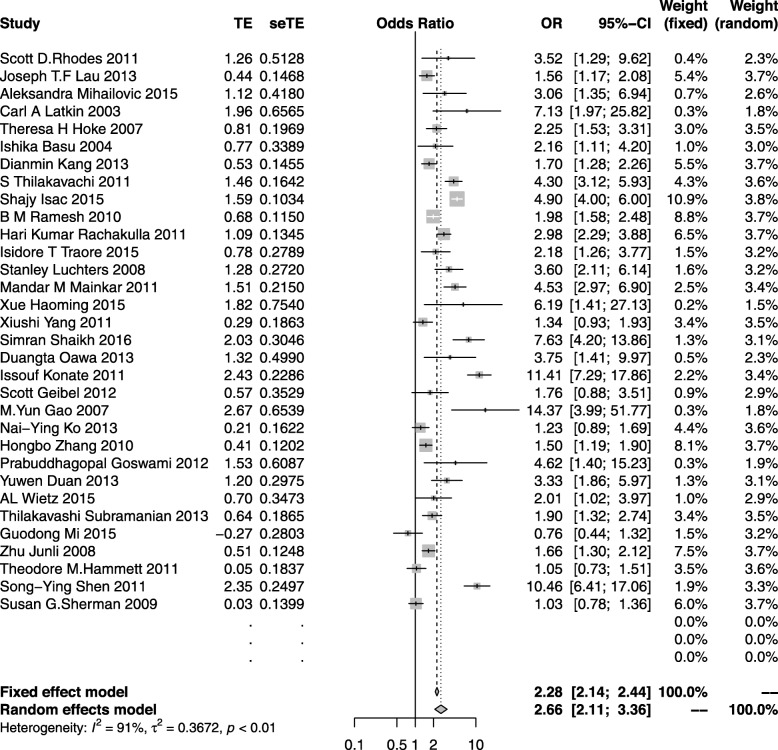
The forest plot of the condom use

Thirty-two studies [19, 22, 31–34, 36–41, 51, 53, 56, 63, 65, 66, 68–80] reported a condom use outcome after intervention with a population of 46,130. Results across these studies were mixed but most revealed an increase in condom use, and only six of the 32 studies showed insignificant condom use after intervention. In general, after the peer education intervention, condom use among the HIV risk population increased with a combined OR of 2.66 (95%CI: 2.11–3.36; *I*^*2*^ = 90%) (Fig. 5). Subgroup analyses also demonstrated significant results among FSWs, MSM and IDUs, with a pooled OR effect of 3.19 (95%CI: 2.41–4.23; *I*^*2*^ = 88%), 1.76 (95%CI:1.37–2.26; *I*^*2*^ = 72%) and 2.84 (95%CI: 1.08–7.48; *I*^*2*^ = 95) respectively, which indicated a positive effect of peer education on condom use (Supplemental figure 9, 10, 11).

Subgroup analyses were also carried out by different partner types and using patterns, revealing that peer-led intervention increased condom use both with casual sexual partners (OR: 2.79; 95%CI: 2.13–3.66; *I*^*2*^ = 84%) and regular sexual partners (OR: 2.45; 95%CI: 1.64–3.66; *I*^*2*^ = 95%). Considering that consistent condom use had a more profound impact on preventing HIV, we also conducted a meta-analysis and found it increased after peer education (OR: 1.80; 95%CI: 1.47–2.21; *I*^*2*^ = 86%) (Supplemental figure 12, 13, 14).

#### HIV measures

**Fig. 6:**
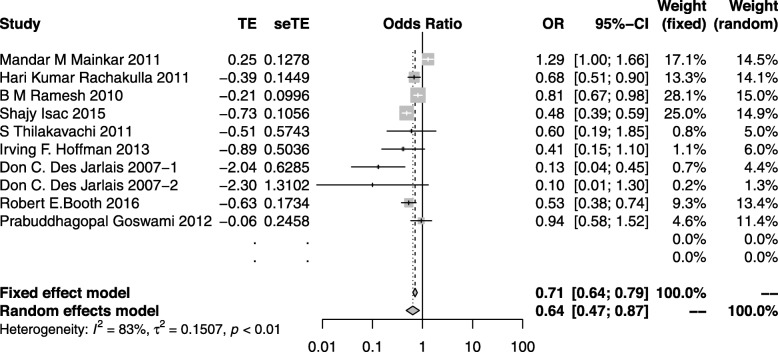
The forest plot of the HIV prevalence

Nine studies [37, 48, 60, 64, 69, 70, 72, 73] generated 10 independent effect sizes on the HIV measure with a population of 28,061. Five showed an insignificant reduction in HIV measure after prevention, and one study found an increased odds of HIV infection. However, the overall results of the meta-analysis suggested 36% lower odds of HIV measure in high risk groups (OR: 0.64; 95%CI: 0.47–0.87; *I*^*2*^ = 83%) (Fig. 6).

### Duration effect of peer education

**Fig. 7:**
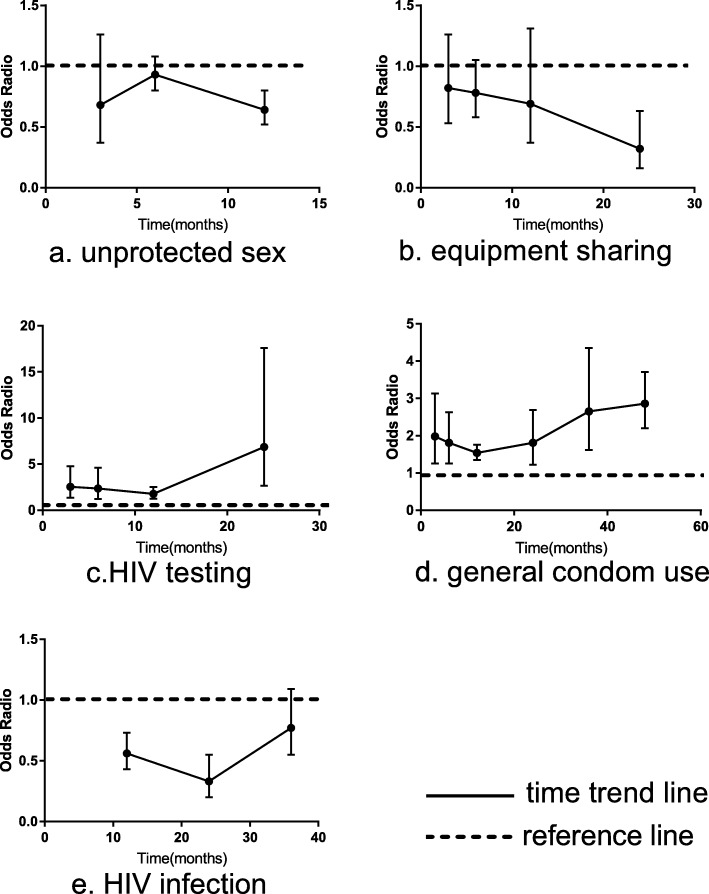
Time trend of peer education effectiveness among HIV high risk groups

Figure 7 presents the effectiveness of peer education among high risk groups for the five outcomes at different time periods.

#### Unprotected sex

The follow-up time of the articles reporting the outcome of unprotected sex was mainly within 12 months of peer education, thus we only analyzed the time effect of unprotected sex at 3, 6, and 12 months. The results highlighted a non-significant effect after 3 and 6 months, with a pooled OR of 0.68 (95% CI:0.37–1.26) and 0.93 (95% CI:0.80–1.08) respectively. However, a significant effect was found after 12 months of peer education, justifying its ability to reduce the cases of unprotected sex in the long term (OR:0.64; 95% CI:0.52–0.80).

#### Equipment sharing

Peer education had a non-significant impact on reducing equipment sharing behaviors within 12 months however the pooled odds ratio showed a downward trend. After 24 months of intervention, the combined effect was 0.32 (95%CI: 0.16–0.63), suggesting that peer education was still valid in reducing equipment sharing over a long period.

#### HIV testing

The persistent effect of peer education on HIV testing was significant with an overall OR of 6.85 after 24 months of intervention, higher than the effects after 3,6,12 months of 2.54 (95%CI: 1.35–4.78), 2.36 (95%CI: 1.21–4.60), 1.78 (95%CI: 1.26–2.52) collectively. The general increasing trend indicated that peer education had a persistent positive impact on encouraging high risk groups to get an HIV test.

#### D. Condom use

The overall time effect of condom use was positive but variable. During the first year after the intervention, the significant impact decreased with a pooled OR of 1.98 (95%CI:1.25–3.13), 1.81 (95%CI:1.25–2.63), 1.54 (95%CI:1.35–1.76) after 3, 6,12 months respectively. However after 12 months, the effect began to increase from 1.81 (95%CI:1.22–2.69) in 24 months to 2.65 (95%CI:1.62–4.35) in 36 months, and finally reaching 2.86 (95%CI:2.2–3.71) in 48 months, suggesting a general persistent effect of peer education on condom use.

#### E. HIV measure

The follow-up time of the HIV measure in the studies focused mainly on 1 to 3 years, thus so we conducted the time analysis in 12, 24, 36 months. Results indicated that after 12 months and 24 months intervention, HIV measure significantly declined with a pooled effect of 0.56 (95%CI:0.43–0.73) and 0.33 (95%CI:0.20–0.55) respectively. Although it still reduced the HIV measure after 36 months, the intervention effect was not statistically significant (OR:0.77; 95%CI:0.55–1.09), implying that the preventive effect of HIV measure may have a slight decline over time.

### Meta-regression

For the HIV test, equipment sharing and condom use meta analyses whose heterogeneity were over 90%, a multivariate meta regression was conducted. The meta regression model quantified the impact of the follow-up time, study sites and high risk groups. Supplemental Table 3 highlights that the follow-up time was the source of heterogeneity for both the meta analyses of HIV testing and equipment sharing, while the study sites and high risk groups did not show significant heterogeneity among these three meta analyses. Although the heterogeneity in the meta-analysis of condom use was relatively high, we had not found the source of heterogeneity. After adjusting for the impact of follow-up time, the pooled effect of the meta-analysis was still significant, which was shown in the results of the effect of duration.

## Discussion

We conducted a systematic review and meta-analysis by combining 59 studies to examine the effectiveness of peer education and analyzed its long-term effects among high risk HIV groups in both developed and developing countries.

Our findings revealed that peer education can effectively promote HIV testing, condom use and reduce unprotected sex and HIV measure among MSM, IDUs and FSWs. The time trend analysis has indicated that this intervention can transform the behaviors consistently with significant results for over maximum 24 months. Moreover, peer education had a more significant effect in developing countries than developed countries, indicating that peer education may be particularly suitable in low and middle-income countries.

Our review is consistent with prior meta analyses showing the positive impacts of peer-based intervention for HIV prevention. In line with our results, a meta-analysis conducted in developing countries demonstrated that peer-led interventions could improve condom use with a combined OR of 1.92 among the general population [8]. However, our study indicated a stronger effect with a combined OR of 2.66, mainly because our target was high risk HIV groups who may involve in more sexual activities. Another meta-analysis showed that the odds of undergoing tests for HIV among MSM who were engaged in peer-led intervention were twice as high as counterparts who were not: the authors’ results were thus almost the same as our analysis with a combined OR of 2.75 on HIV testing behavior [23]. A previous systematic review found a 32% reduction in unprotected sex for group-based interventions among MSM [27], slightly higher than our study (24%), which indicated a greater effect among MSM on unprotected sex behavior. We thus believe that our systematic review and meta-analysis contributes to the growing body of work on the utility of peer-led intervention by adding evidence for the critical outcome among high risk HIV groups worldwide.

Our subgroup analyses revealed that peer education has a significant effect on increasing condom use both with casual partners (OR = 2.79) and regular partners (OR = 2.45) as well as on consistent condom use (OR = 1.80). Condom use has been proven to effectively prevent transmission among high risk HIV groups [66], especially among FSWs. Their regular partners and clients include different types of men, from “boyfriends” and sex work venue managers to “protectors” who prevent the women from being assaulted at night [81]. The HIV risk is probably high among these men who have many sex worker girlfriends [65]. From our findings, we strongly believe that the continuum of peer education was crucial in obtaining a high adherence level of FSWs to use condom with diverse partners. Nevertheless, a recent study by Karnataka suggested that if an FSW started sex work and subsequently acquires a regular sex partner, condoms were more likely to be used, compared with situations where marriage occurs first and sex work begins later on [82]. Thus more work is required to understand and mitigate sexual risk and to increase condom use among high risk HIV groups.

We believe that the greatest strength of this study is that we analyzed the time trend of peer education effectiveness among high risk HIV groups. Time analysis verified that peer education has a consistent effect on changing behaviors and reducing HIV incidence over 24 months, although with some fluctuations. The results of HIV testing and condom use were significant overall with a gradual increasing trend, indicating a persistent and positive effect on changing these two behaviors. As regards equipment sharing and unprotected sex, peer education might be able to reduce the occurrence rate significantly after 12 months. The effect became gradually robust which was in accordance with the HIV testing and condom use results. Stages of change (SOCs) revealed that people move through a series of stages when modifying behaviors [83, 84]. The transtheoretical model (TTM) posits that people move through five specific SOCs when changing health behaviors: precontemplation, contemplation, preparation, action, and maintenance [85]. It is therefore normal that the outcomes were not completely clearly or even reversed during the behavior change process.

In terms of HIV infection, which included the time periods of 12, 24, 36 months, the significant results maintained, however the effects weakened at 36 months. There are other factors that may lead to HIV infection, such as economic pressures driving [66], the risk social environment [85], severe stigmatization and discrimination. Other factors, such as the increasing registration for anti-retroviral treatment (ART) among adults and women, and the misconception that people on ART do not transmit HIV, could have contributed to newly-infected HIV cases [70]. Additional research is needed to identify the structural characteristics of the social networks of high-risk groups that may facilitate more successful peer education intervention.

Our review also has several limitations. First, we found evidence of publication bias in the meta-analysis of HIV testing and unprotected sex. Disproportionate reporting of significant associations in published work can result in an overestimate of the impact of peer education [86]. Therefore, we conducted sensitivity analyses and subgroup analyses in an attempt to reduce biases. Second, when analyzing the time trend of peer education effectiveness, biases might exist in studies which were conducted over a period of 12 months since they were mostly serial cross sectional studies. However, the sample size of those studies was sufficiently large to minimize biases as much as possible. Finally, the heterogeneity across studies was significant, which may overstate the pooled estimates. Considering that subgroup analyses and meta-regression were conducted, we believe that our results are still reliable.

## Conclusion

In conclusion, peer education can effectively change behaviors and reduce HIV measure among high risk HIV groups. More importantly, it can maintain its effects consistently with significant results over 24 months period. Nevertheless, further RCT studies with longer follow-up period are still needed to precisely validate the effect of duration.^]^ In order to significantly reduce the prevalence of HIV, peer education characterized by its low-costing features tend to particularly beneficial for low- and middle-income countries and should be promoted widely in resource-limited regions.

## Supplementary information

**Additional file 1.** The information of the selected studies.

**Additional file 2: Table S1.** Study quality assessment. **Table S2.** The results of egger test.

**Additional file 3: Figure S1.** The sensitivity analysis of the unprotected sex. **Figure S2.** The sensitivity analysis of the HIV testing. **Figure S3.** The sensitivity analysis of the equipment use. **Figure S** The sensitivity analysis of the HIV prevalence. **Figure S5.** The sensitivity analysis of the general condom use. **Figure S6.** The sensitivity analysis of the consistent condom use. **Figure S7.** The sensitivity analysis of the condom use with casual partners. **Figure S8.** The sensitivity analysis of the condom use with regular partners.

**Additional file 4: Figure S9.** The forest plot of the condom use among FSWs. **Figure S10.** The forest plot of the condom use among IDUs. **Figure S11.** The forest plot of the condom use among MSM. **Figure S12.** The forest plot of condom use with casual partners. **Figure S13.** The forest plot of condom use with regular partners. **Figure S14.** The forest plot of consistent condom use.

## Data Availability

All data generated or analyzed during this study are included in this published article and its supplementary information files.

## References

[1] Solomon SS, Solomon S, McFall AM, Srikrishnan AK, Anand S, Verma V, Vasudevan CK, Balakrishnan P, Ogburn EL, Moulton LH (2019). Integrated HIV testing, prevention, and treatment intervention for key populations in India: a cluster-randomised trial. Lancet Hiv.

[2] Global HIV & AIDS statistics-2019 fact sheet. https://www.unaids.org/en/resources/fact-sheet. Accessed 15 Feb 2020..

[3] Holtz TH, Pattanasin S, Chonwattana W, Tongtoyai J, Chaikummao S, Varangrat A, Mock PA (2015). Longitudinal analysis of key HIV-risk behavior patterns and predictors in men who have sex with men, Bangkok**,** Thailand. Arch Sex Behav.

[4] Kurth AE, Cleland CM, Des Jarlais DC, Musyoki H, Lizcano JA, Chhun N, Cherutich P (2015). HIV prevalence, estimated incidence, and risk behaviors among people who inject drugs in Kenya. J Acquir Immune Defic Syndr.

[5] Burt RD, Tinsley J, Glick SN (2017). A decline in HIV testing among persons who inject drugs in the Seattle area, 2004-2015. J Acquir Immune Defic Syndr.

[6] Johnson WD, Diaz RM, Flanders WD, Goodman M, Hill AN, Holtgrave D, Malow R, McClellan WM (2008). Behavioral interventions to reduce risk for sexual transmission of HIV among men who have sex with men. Cochrane Database Syst Rev.

[7] Herbst JH, Beeker C, Mathew A, McNally T, Passin WF, Kay LS, Crepaz N, Lyles CM, Briss P, Chattopadhyay S (2007). The effectiveness of individual-, group-, and community-level HIV behavioral risk-reduction interventions for adult men who have sex with men: a systematic review. Am J Prev Med.

[8] Medley A, Kennedy C, O'Reilly K, Sweat M (2009). Effectiveness of peer education interventions for HIV prevention in developing countries: a systematic review and meta-analysis. AIDS Educ Prev.

[9] Webel AR (2010). Testing a peer-based symptom management intervention for women living with HIV/AIDS. AIDS Care.

[10] Simoni JM, Nelson KM, Franks JC, Yard SS, Lehavot K (2011). Are peer interventions for HIV efficacious? A systematic review. AIDS Behav.

[11] Campbell C, Mzaidume Z (2001). Grassroots participation, peer education, and HIV prevention by sex workers in South Africa. Am J Public Health.

[12] Yan H, Zhang R, Wei C, Li J, Xu J, Yang H, McFarland W (2014). A peer-led, community-based rapid HIV testing intervention among untested men who have sex with men in China: an operational model for expansion of HIV testing and linkage to care. Sex Transm Infect.

[13] Bagnall AM, South J, Hulme C, Woodall J, Vinall-Collier K, Raine G, Kinsella K, Dixey R, Harris L, Wright NM (2015). A systematic review of the effectiveness and cost-effectiveness of peer education and peer support in prisons. BMC Public Health.

[14] Chola L, Fadnes LT, Engebretsen IM, Nkonki L, Nankabirwa V, Sommerfelt H, Tumwine JK, Tylleskar T, Robberstad B (2015). Cost-effectiveness of peer Counselling for the promotion of exclusive breastfeeding in Uganda. PLoS One.

[15] Panic N, Leoncini E, de Belvis G, Ricciardi W, Boccia S (2013). Evaluation of the endorsement of the preferred reporting items for systematic reviews and meta-analysis (PRISMA) statement on the quality of published systematic review and meta-analyses. PLoS One.

[16] Higgins JP, Thompson SG, Deeks JJ, Altman DG (2003). Measuring inconsistency in meta-analyses. BMJ.

[17] Liu Y, Vermund SH, Ruan Y, et al. Peer counselling versus standard-of-care on reducing high-risk behaviours among newly diagnosed HIV-positive men who have sex with men in Beijing, China: a randomized intervention study. J Int AIDS Soc. 2018;21(2):e25079. 10.1002/jia2.25079 PMC5808102 29430845

[18] Adebajo S, Eluwa G, Njab J, Oginni A, Ukwuije F, Ahonsi B, Lorenc T (2015). Evaluating the effect of HIV prevention strategies on uptake of HIV counselling and testing among male most-at-risk-populations in Nigeria; a cross-sectional analysis. Sex Transm Infect.

[19] Mi G, Wu Z, Wang X, Shi CX, Yu F, Li T, Zhang L, McGoogan JM, Pang L, Xu J (2015). Effects of a quasi-randomized web-based intervention on risk behaviors and treatment seeking among HIV-positive men who have sex with men in Chengdu**,** China. Curr Hiv Res.

[20] Hidalgo MA, Kuhns LM, Hotton AL, Johnson AK, Mustanski B, Garofalo R (2015). The MyPEEPS randomized controlled trial: a pilot of preliminary efficacy, feasibility, and acceptability of a group-level, HIV risk reduction intervention for young men who have sex with men. Arch Sex Behav.

[21] Young SD, Cumberland WG, Nianogo R, Menacho LA, Galea JT, Coates T (2015). The HOPE social media intervention for global HIV prevention in Peru: a cluster randomised controlled trial. Lancet HIV.

[22] Mihailovic A, Tobin K, Latkin C (2015). The influence of a peer-based HIV prevention intervention on conversation about HIV prevention among people who inject drugs in Baltimore, Maryland. Aids Behav.

[23] Shangani S, Escudero D, Kirwa K, Harrison A, Marshall B, Operario D (2017). Effectiveness of peer-led interventions to increase HIV testing among men who have sex with men: a systematic review and meta-analysis. AIDS Care.

[24] Wong T, Pharr JR, Bungum T, Coughenour C, Lough NL (2019). Effects of peer sexual health education on college campuses: a systematic review. Health Promot Pract.

[25] Hu J, Wang X, Guo S, Chen F, Wu YY, Ji FJ, Fang X (2019). Peer support interventions for breast cancer patients: a systematic review. Breast Cancer Res Treat.

[26] Rose-Clarke K, Bentley A, Marston C, Prost A (2019). Peer-facilitated community-based interventions for adolescent health in low- and middle-income countries: a systematic review. PLoS One.

[27] Ye S, Yin L, Amico R, Simoni J, Vermund S, Ruan Y, Shao Y, Qian HZ (2014). Efficacy of peer-led interventions to reduce unprotected anal intercourse among men who have sex with men: a meta-analysis. PLoS One.

[28] Egger M, Davey SG, Schneider M, Minder C (1997). Bias in meta-analysis detected by a simple, graphical test. BMJ.

[29] Davey-Rothwell MA, Tobin K, Yang C, Sun CJ, Latkin CA (2011). Results of a randomized controlled trial of a peer Mentor HIV/STI prevention intervention for women over an 18 month follow-up. AIDS Behav.

[30] Surratt HL, O Grady C, Kurtz SP, Levi-Minzi MA, Chen M (2014). Outcomes of a behavioral intervention to reduce HIV risk among drug-involved female sex workers. AIDS Behav.

[31] Rhodes SD, McCoy TP, Vissman AT, DiClemente RJ, Duck S, Hergenrather KC, Foley KL, Alonzo J, Bloom FR, Eng E (2011). A randomized controlled trial of a culturally congruent intervention to increase condom use and HIV testing among heterosexually active immigrant Latino men. AIDS Behav.

[32] Lau JT, Tsui HY, Lau MM (2013). A pilot clustered randomized control trial evaluating the efficacy of a network-based HIV peer-education intervention targeting men who have sex with men in Hong Kong, China. Aids Care.

[33] Wirtz AL, Trapence G, Jumbe V, Umar E, Ketende S, Kamba D, Berry M, Strömdahl S, Beyrer C, Muula AS (2015). Feasibility of a combination HIV prevention program for men who have sex with men in Blantyre, Malawi. JAIDS.

[34] Ko NY, Hsieh CH, Wang MC, Lee C, Chen CL, Chung AC, Hsu ST (2013). Effects of internet popular opinion leaders (iPOL) among internet-using men who have sex with men. J Med Internet Res.

[35] Young SD, Cumberland WG, Lee S, Jaganath D, Szekeres G, Coates T (2013). Social networking technologies as an emerging tool for HIV prevention. Ann Intern Med.

[36] Yuwen Duan HZJW (2013). Community-based peer intervention to reduce HIV risk among men who have sex with men in Sichuan province, China. Aids Educ Prev.

[37] Goswami P, Rachakulla HK, Ramakrishnan L, Mathew S, Ramanathan S, George B, Adhikary R, Kodavalla V, Rajkumar H, Paranjape RS (2013). An assessment of a large-scale HIV prevention programme for high-risk men who have sex with men and transgenders in Andhra Pradesh, India: using data from routine programme monitoring and repeated cross-sectional surveys. BMJ Open.

[38] Subramanian T, Ramakrishnan L, Aridoss S, Goswami P, Kanguswami B, Shajan M, Adhikary R, Purushothaman GKC, Ramamoorthy SK, Chinnaswamy E (2013). Increasing condom use and declining STI prevalence in high-risk MSM and TGs: evaluation of a large-scale prevention program in Tamil Nadu, India. Bmc Public Health.

[39] Zhang H, Wu Z, Zheng Y, Wang J, Zhu J, Xu J (2010). A pilot intervention to increase condom use and HIV testing and counseling among men who have sex with men in Anhui, China. J Acquir Immune Defic Syndr.

[40] Jun-li Z, Hong-bo Z, Zun-you WU, Ying-jun Z, Juan XU, Jun W, Hong-hua WU, Lin C, Der GJ (2008). HIV risk behavior based on intervention among men who have sex with men peer groups in Anhui province. Chin J Prev Med.

[41] Yun Gao M, Wang S (2007). Participatory communication and HIV/AIDS prevention in a Chinese marginalized (MSM) population. AIDS Care.

[42] Amirkhanian YA, Kelly JA, Kabakchieva E, Kirsanova AV, Vassileva S, Takacs J, DiFranceisco WJ, McAuliffe TL, Khoursine RA, Mocsonaki L (2005). A randomized social network HIV prevention trial with young men who have sex with men in Russia and Bulgaria. AIDS (London, England).

[43] Williamson LM, Hart GJ, Flowers P, Frankis JS, Der GJ (2001). The gay Men's task force: the impact of peer education on the sexual health behaviour of homosexual men in Glasgow. Sex Transm Infect.

[44] Theall KP, Fleckman J, Jacobs M (2015). Impact of a community popular opinion leader intervention among African American adults in a southeastern United States community. AIDS Educ Prev.

[45] Jain B, Krishnan S, Ramesh S, Sabarwal S, Garg V, Dhingra N (2014). Effect of peer-led outreach activities on injecting risk behavior among male drug users in Haryana**,** India. Harm Reduct J.

[46] Go VF, Frangakis C, Le Minh N, Latkin CA, Ha TV, Mo TT, Sripaipan T, Davis W, Zelaya C, Vu PT (2013). Effects of an HIV peer prevention intervention on sexual and injecting risk behaviors among injecting drug users and their risk partners in Thai Nguyen, Vietnam: a randomized controlled trial. Soc Sci Med.

[47] Latkin C, Donnell D, Liu T, Davey-Rothwell M, Celentano D, Metzger D (2013). The dynamic relationship between social norms and behaviors: the results of an HIV prevention network intervention for injection drug users. Addiction.

[48] Hoffman IF, Latkin CA, Kukhareva PV, Malov SV, Batluk JV, Shaboltas AV, Skochilov RV, Sokolov NV, Verevochkin SV, Hudgens MG (2013). A peer-educator network HIV prevention intervention among injection drug users: results of a randomized controlled trial in St. Petersburg, Russia. AIDS Behav.

[49] Mackesy-Amiti ME, Finnegan L, Ouellet LJ, Golub ET, Hagan H, Hudson SM, Latka MH, Garfein RS (2013). Peer-education intervention to reduce injection risk behaviors benefits high-risk Young injection drug users: a latent transition analysis of the CIDUS 3/DUIT study. AIDS Behav.

[50] Hammett TM, Des Jarlais DC, Kling R, Kieu BT, McNicholl JM, Wasinrapee P, McDougal JS, Liu W, Chen Y, Meng D (2012). Controlling HIV epidemics among injection drug users: eight years of cross-border HIV prevention interventions in Vietnam and China. PLoS One.

[51] Hammett TM, Kling R, Van NTH, Son DH, Binh KT, Oanh KTH (2012). HIV prevention interventions for female sexual Partners of Injection Drug Users in Hanoi, Vietnam: 24-month evaluation results. AIDS Behav.

[52] Tobin KE, Kuramoto SJ, Davey-Rothwell MA, Latkin CA (2011). The STEP into action study: a peer-based, personal risk network-focused HIV prevention intervention with injection drug users in Baltimore**,** Maryland. Addiction.

[53] Shen S, Zhang Z, Tucker JD, Chang H, Zhang G, Lin A (2011). Peer-based behavioral health program for drug users in China: a pilot study. BMC Public Health.

[54] Booth RE, Lehman WE, Latkin CA, Dvoryak S, Brewster JT, Royer MS, Sinitsyna L (2011). Individual and network interventions with injection drug users in 5 Ukraine cities. Am J Public Health.

[55] Latkin CA, Donnell D, Metzger D, Sherman S, Aramrattna A, Davis-Vogel A, Quan VM, Gandham S, Vongchak T, Perdue T (2009). The efficacy of a network intervention to reduce HIV risk behaviors among drug users and risk partners in Chiang Mai, Thailand and Philadelphia**,** USA. Soc Sci Med.

[56] Purcell DW, Latka MH, Metsch LR, et al. Results from a randomized controlled trial of a peer-mentoring intervention to reduce HIV transmission and increase access to care and adherence to HIV medications among HIV-seropositive injection drug users. J Acquir Immune Defic Syndr. 2007;46 Suppl 2:S35–47. 10.1097/QAI.0b013e31815767c4 18089983

[57] Weeks MR, Li J, Dickson-Gomez J, Convey M, Martinez M, Radda K, Clair S (2009). Outcomes of a peer HIV prevention program with injection drug and crack users: the risk avoidance partnership. Subst Use Misuse.

[58] Purcell DW, Latka MH, Metsch LR, Latkin CA, Gómez CA, Mizuno Y, Arnsten JH, Wilkinson JD, Knight KR, Knowlton AR *et al*: Results From a Randomized Controlled Trial of a Peer-Mentoring Intervention to Reduce HIV Transmission and Increase Access to Care and Adherence to HIV Medications Among HIV-Seropositive Injection Drug Users. JAIDS 2007, 46 Suppl 2, HIV prevention and clinical care for HIV-positive injection drug users: lessons from the INSPIRE study (supplement 2):S35-S47. 10.1097/QAI.0b013e31815767c4 18089983

[59] Garfein RS, Golub ET, Greenberg AE, Hagan H, Hanson DL, Hudson SM, Kapadia F, Latka MH, Ouellet LJ, Purcell DW (2007). A peer-education intervention to reduce injection risk behaviors for HIV and hepatitis C virus infection in young injection drug users. AIDS (London, England).

[60] Des Jarlais DC, Kling R, Hammett TM, Ngu D, Liu W, Chen Y, Binh KT, Friedmann P (2007). Reducing HIV infection among new injecting drug users in the China-Vietnam cross border project. AIDS.

[61] Broadhead RS, Volkanevsky VL, Rydanova T, Ryabkova M, Borch C, van Hulst Y, Fullerton A, Sergeyev B, Heckathorn DD (2006). Peer-driven HIV interventions for drug injectors in Russia: first year impact results of a field experiment. INT J DRUG POLICY.

[62] Hammett TM (2005). Community Attitudes Toward HIV Prevention for Injection Drug Users: Findings from a Cross-Border Project in Southern China and Northern Vietnam. J Urban Health.

[63] Latkin CA, Sherman S, Knowlton A (2003). HIV prevention among drug users: outcome of a network-oriented peer outreach intervention. Health Psychol.

[64] Booth RE, Davis JM, Dvoryak S, Brewster JT, Lisovska O, Strathdee SA, Latkin CA (2016). HIV incidence among people who inject drugs (PWIDs) in Ukraine: results from a clustered randomised trial. The Lancet HIV.

[65] Traore IT, Meda N, Hema NM, Ouedraogo D, Some F, Some R, Niessougou J, Sanon A, Konate I, Van De Perre P (2015). HIV prevention and care services for female sex workers: efficacy of a targeted community-based intervention in Burkina Faso. J Int AIDS Soc.

[66] Kang D, Tao X, Liao M, Li J, Zhang N, Zhu X, Sun X, Lin B, Su S, Hao L (2013). An integrated individual, community, and structural intervention to reduce HIV/STI risks among female sex workers in China. BMC Public Health.

[67] Ang A, Morisky DE (2012). A multilevel analysis of the impact of socio-structural and environmental influences on condom use among female sex workers. AIDS Behav.

[68] Xiushi Yang GXXL (2011). The efficacy of a peer-assisted multi-component behavioral intervention among female entertainment workers in China: an initial assessment. AIDS Care.

[69] Thilakavathi S, Boopathi K, Girish Kumar CP, Santhakumar A, Senthilkumar R, Eswaramurthy C, Ilaya Bharathy V, Ramakrishnan L, Thongamba G, Adhikary R (2011). Assessment of the scale, coverage and outcomes of the Avahan HIV prevention program for female sex workers in Tamil Nadu, India: is there evidence of an effect?. Bmc Public Health.

[70] Rachakulla HK, Kodavalla V, Rajkumar H, Prasad SPV, Kallam S, Goswami P, Dale J, Adhikary R, Paranjape R, Brahmam GNV (2011). Condom use and prevalence of syphilis and HIV among female sex workers in Andhra Pradesh, India - following a large-scale HIV prevention intervention. Bmc Public Health.

[71] Konate I, Traore L, Ouedraogo A, Sanon A, Diallo R, Ouedraogo JL, Huet C, Millogo I, Andonaba JB, Mayaud P (2011). Linking HIV prevention and care for community interventions among high-risk women in Burkina Faso--the ARNS 1222 “Yerelon” cohort. J Acquir Immune Defic Syndr.

[72] Mainkar MM, Pardeshi DB, Dale J, Deshpande S, Khazi S, Gautam A, Goswami P, Adhikary R, Ramanathan S, George B (2011). Targeted interventions of the Avahan program and their association with intermediate outcomes among female sex workers in Maharashtra, India. Bmc Public Health.

[73] Ramesh BM, Beattie TSH, Shajy I, Washington R, Jagannathan L, Reza-Paul S, Blanchard JF, Moses S (2010). Changes in risk behaviours and prevalence of sexually transmitted infections following HIV preventive interventions among female sex workers in five districts in Karnataka state, South India. Sex Transm Infect.

[74] Luchters S, Chersich MF, Rinyiru A, Barasa MS, King'Ola N, Mandaliya K, Bosire W, Wambugu S, Mwarogo P, Temmerman M (2008). Impact of five years of peer-mediated interventions on sexual behavior and sexually transmitted infections among female sex workers in Mombasa**,** Kenya. Bmc Public Health.

[75] Xue H, Luo Z, Zhu Z, Yang X, Yang L, Yang J, Duo L, Liu W (2015). Intervention caused changes in high risk sex behaviors among female sex workers from Vietnam in Yunnan, 2009-2013. Zhonghua Liu Xing Bing Xue Za Zhi.

[76] Geibel S, King'Ola N, Temmerman M, Luchters S (2012). The impact of peer outreach on HIV knowledge and prevention behaviours of male sex workers in Mombasa**,** Kenya. Sex Transm Infect.

[77] Hoke TH, Feldblum PJ, Damme KV, Nasution MD, Grey TW, Wong EL, Ralimamonjy L, Raharimalala L, Rasamindrakotroka A (2007). Randomised controlled trial of alternative male and female condom promotion strategies targeting sex workers in Madagascar. Sex Transm Infect.

[78] Ishika Basu SJMJ (2004). HIV prevention among sex Workers in India. J Acquir Immune Defic Syndr.

[79] Shaikh S, Mburu G, Arumugam V, Mattipalli N, Aher A, Mehta S, Robertson J (2016). Empowering communities and strengthening systems to improve transgender health: outcomes from the Pehchan programme in India. J Int Aids Soc.

[80] Pawa D, Firestone R, Ratchasi S, Dowling O, Jittakoat Y, Duke A, Mundy G. Reducing HIV risk among transgender women in Thailand: a quasi-experimental evaluation of the sisters program. PLoS One. 2013;8(10):e77113. 10.1371/journal.pone.0077113 PMC3812213 24204750

[81] Wang C, Hawes SE, Gaye A, Sow PS, Ndoye I, Manhart LE, Wald A, Critchlow CW, Kiviat NB (2007). HIV prevalence, previous HIV testing, and condom use with clients and regular partners among Senegalese commercial sex workers. Sex Transm Infect.

[82] Isac S, Prakash R, Ramesh BM, et al. Challenges in increasing condom use among female sex workers with their regular sex partners in Karnataka state, South India. Washington DC: International AIDS Conference; 2012.

[83] Helitzer DL, Peterson AB, Sanders M, Thompson J (2007). Relationship of stages of change to attendance in a diabetes prevention program. Am J Health Promot.

[84] Prochaska JO, DiClemente CC (1983). Stages and processes of self-change of smoking: toward an integrative model of change. J Consult Clin Psychol.

[85] Tseng HM, Liao SF, Wen YP, Chuang YJ (2017). Stages of change concept of the transtheoretical model for healthy eating links health literacy and diabetes knowledge to glycemic control in people with type 2 diabetes. Prim Care Diabetes.

[86] Yuan T, Fitzpatrick T, Ko NY, Cai Y, Chen Y, Zhao J, Li L, Xu J, Gu J, Li J (2019). Circumcision to prevent HIV and other sexually transmitted infections in men who have sex with men: a systematic review and meta-analysis of global data. Lancet Glob Health.

