# Supplementary material for: Peer education for HIV prevention among high-risk groups: a systematic review and meta-analysis
Source: BMC Infect Dis. 2020 May 12;20:338. doi: 10.1186/s12879-020-05003-9 (PMC7218508; doi:10.1186/s12879-020-05003-9)
Supplement: Supplementary file 1 — Additional file 1. The information of the selected studies. [file 12879_2020_5003_MOESM1_ESM.docx]

**Selected Studies**

1. Liu Y, Vermund SH, Ruan Y, Liu H, Rivet AK, Simoni JM, Shepherd BE, Shao Y, Qian HZ: **Peer counselling versus standard-of-care on reducing high-risk behaviours among newly diagnosed HIV-positive men who have sex with men in Beijing, China: a randomized intervention study**. *J INT AIDS SOC* 2018, **21**(2).

2. Adebajo S, Eluwa G, Njab J, Oginni A, Ukwuije F, Ahonsi B, Lorenc T: **Evaluating the effect of HIV prevention strategies on uptake of HIV counselling and testing among male most-at-risk-populations in Nigeria; a cross-sectional analysis**. *SEX TRANSM INFECT* 2015, **91**(8):555-560.

3. Mi G, Wu Z, Wang X, Shi CX, Yu F, Li T, Zhang L, McGoogan JM, Pang L, Xu J *et al*: **Effects of a Quasi-Randomized Web-Based Intervention on Risk Behaviors and Treatment Seeking Among HIV-Positive Men Who Have Sex With Men in Chengdu, China**. *CURR HIV RES* 2015, **13**(6):490-496.

4. Hidalgo MA, Kuhns LM, Hotton AL, Johnson AK, Mustanski B, Garofalo R: **The MyPEEPS randomized controlled trial: a pilot of preliminary efficacy, feasibility, and acceptability of a group-level, HIV risk reduction intervention for young men who have sex with men**. *ARCH SEX BEHAV* 2015, **44**(2):475-485.

5. Young SD, Cumberland WG, Nianogo R, Menacho LA, Galea JT, Coates T: **The HOPE social media intervention for global HIV prevention in Peru: a cluster randomised controlled trial**. *The Lancet HIV* 2015, **2**(1):e27-e32.

6. Lau JT, Tsui HY, Lau MM: **A pilot clustered randomized control trial evaluating the efficacy of a network-based HIV peer-education intervention targeting men who have sex with men in Hong Kong, China**. *AIDS CARE* 2013, **25**(7):812-819.

7. Wirtz AL, Trapence G, Jumbe V, Umar E, Ketende S, Kamba D, Berry M, Strömdahl S, Beyrer C, Muula AS *et al*: **Feasibility of a Combination HIV Prevention Program for Men Who Have Sex With Men in Blantyre, Malawi**. *JAIDS Journal of Acquired Immune Deficiency Syndromes* 2015, **70**(2):155-162.

8. Yan H, Zhang R, Wei C, Li J, Xu J, Yang H, McFarland W: **A peer-led, community-based rapid HIV testing intervention among untested men who have sex with men in China: an operational model for expansion of HIV testing and linkage to care**. *SEX TRANSM INFECT* 2014, **90**(5):388-393.

9. Ko NY, Hsieh CH, Wang MC, Lee C, Chen CL, Chung AC, Hsu ST: **Effects of Internet popular opinion leaders (iPOL) among Internet-using men who have sex with men**. *J MED INTERNET RES* 2013, **15**(2):e40.

10. Young SD, Cumberland WG, Lee S, Jaganath D, Szekeres G, Coates T: **Social Networking Technologies as an Emerging Tool for HIV Prevention**. *ANN INTERN MED* 2013, **159**(5):318.

11. Yuwen Duan HZJW: **Community-based peer intervention to reduce HIV risk among men who have sex with men in Sichuan province, China**. *AIDS EDUC PREV* 2013, **1**(25):38-48.

12. Goswami P, Rachakulla HK, Ramakrishnan L, Mathew S, Ramanathan S, George B, Adhikary R, Kodavalla V, Rajkumar H, Paranjape RS *et al*: **An assessment of a large-scale HIV prevention programme for high-risk men who have sex with men and transgenders in Andhra Pradesh, India: using data from routine programme monitoring and repeated cross-sectional surveys**. *BMJ OPEN* 2013, **3**(4):e2183.

13. Subramanian T, Ramakrishnan L, Aridoss S, Goswami P, Kanguswami B, Shajan M, Adhikary R, Purushothaman GKC, Ramamoorthy SK, Chinnaswamy E *et al*: **Increasing condom use and declining STI prevalence in high-risk MSM and TGs: evaluation of a large-scale prevention program in Tamil Nadu, India**. *BMC PUBLIC HEALTH* 2013, **13**(1):857.

14. Zhang H, Wu Z, Zheng Y, Wang J, Zhu J, Xu J: **A pilot intervention to increase condom use and HIV testing and counseling among men who have sex with men in Anhui, China**. *J Acquir Immune Defic Syndr* 2010, **53 Suppl 1**:S88-S92.

15. Jun-li Z, Hong-bo Z, Zun-you WU, Ying-jun Z, Juan XU, Jun W, Hong-hua WU, Lin C, Der GJ: **HIV risk behavior based on intervention among men who have sex with men peer groups in Anhui province**. *CHINESE JOURNAL OF PREVENTIVE MEDICINE* 2008, **42**(12):895-900.

16. Yun Gao M, Wang S: **Participatory communication and HIV/AIDS prevention in a Chinese marginalized (MSM) population**. *AIDS CARE* 2007, **19**(6):799-810.

17. Amirkhanian YA, Kelly JA, Kabakchieva E, Kirsanova AV, Vassileva S, Takacs J, DiFranceisco WJ, McAuliffe TL, Khoursine RA, Mocsonaki L: **A randomized social network HIV prevention trial with young men who have sex with men in Russia and Bulgaria**. *AIDS (London, England)* 2005, **19**(16):1897-1905.

18. Williamson LM, Hart GJ, Flowers P, Frankis JS, Der GJ: **The Gay Men's Task Force: the impact of peer education on the sexual health behaviour of homosexual men in Glasgow**. *SEX TRANSM INFECT* 2001, **77**(6):427-432.

19. Mihailovic A, Tobin K, Latkin C: **The influence of a peer-based HIV prevention intervention on conversation about HIV prevention among people who inject drugs in Baltimore, Maryland**. *AIDS BEHAV* 2015, **19**(10):1792-1800.

20. Theall KP, Fleckman J, Jacobs M: **Impact of a community popular opinion leader intervention among African American adults in a southeastern United States community**. *AIDS education and prevention : official publication of the International Society for AIDS Education* 2015, **27**(3):275-287.

21. Jain B, Krishnan S, Ramesh S, Sabarwal S, Garg V, Dhingra N: **Effect of peer-led outreach activities on injecting risk behavior among male drug users in Haryana, India**. *HARM REDUCT J* 2014, **11**:3.

22. Go VF, Frangakis C, Le Minh N, Latkin CA, Ha TV, Mo TT, Sripaipan T, Davis W, Zelaya C, Vu PT *et al*: **Effects of an HIV peer prevention intervention on sexual and injecting risk behaviors among injecting drug users and their risk partners in Thai Nguyen, Vietnam: A randomized controlled trial**. *SOC SCI MED* 2013, **96**:154-164.

23. Latkin C, Donnell D, Liu T, Davey-Rothwell M, Celentano D, Metzger D: **The dynamic relationship between social norms and behaviors: the results of an HIV prevention network intervention for injection drug users**. *ADDICTION* 2013, **108**(5):934-943.

24. Hoffman IF, Latkin CA, Kukhareva PV, Malov SV, Batluk JV, Shaboltas AV, Skochilov RV, Sokolov NV, Verevochkin SV, Hudgens MG *et al*: **A Peer-Educator Network HIV Prevention Intervention Among Injection Drug Users: Results of a Randomized Controlled Trial in St. Petersburg, Russia**. *AIDS BEHAV* 2013, **17**(7):2510-2520.

25. Mackesy-Amiti ME, Finnegan L, Ouellet LJ, Golub ET, Hagan H, Hudson SM, Latka MH, Garfein RS: **Peer-Education Intervention to Reduce Injection Risk Behaviors Benefits High-Risk Young Injection Drug Users: A Latent Transition Analysis of the CIDUS 3/DUIT Study**. *AIDS BEHAV* 2013, **17**(6):2075-2083.

26. Hammett TM, Des Jarlais DC, Kling R, Kieu BT, McNicholl JM, Wasinrapee P, McDougal JS, Liu W, Chen Y, Meng D *et al*: **Controlling HIV epidemics among injection drug users: eight years of Cross-Border HIV prevention interventions in Vietnam and China**. *PLOS ONE* 2012, **7**(8):e43141.

27. Hammett TM, Kling R, Van NTH, Son DH, Binh KT, Oanh KTH: **HIV Prevention Interventions for Female Sexual Partners of Injection Drug Users in Hanoi, Vietnam: 24-Month Evaluation Results**. *AIDS BEHAV* 2012, **16**(5):1164-1172.

28. Tobin KE, Kuramoto SJ, Davey-Rothwell MA, Latkin CA: **The STEP into Action study: a peer-based, personal risk network-focused HIV prevention intervention with injection drug users in Baltimore, Maryland**. *ADDICTION* 2011, **106**(2):366-375.

29. Shen S, Zhang Z, Tucker JD, Chang H, Zhang G, Lin A: **Peer-based behavioral health program for drug users in China: a pilot study**. *BMC PUBLIC HEALTH* 2011, **11**(1

):693.

30. Booth RE, Lehman WE, Latkin CA, Dvoryak S, Brewster JT, Royer MS, Sinitsyna L: **Individual and network interventions with injection drug users in 5 Ukraine cities**. *AM J PUBLIC HEALTH* 2011, **101**(2):336-343.

31. Latkin CA, Donnell D, Metzger D, Sherman S, Aramrattna A, Davis-Vogel A, Quan VM, Gandham S, Vongchak T, Perdue T *et al*: **The efficacy of a network intervention to reduce HIV risk behaviors among drug users and risk partners in Chiang Mai, Thailand and Philadelphia, USA**. *SOC SCI MED* 2009, **68**(4):740-748.

32. Sherman SG, Sutcliffe C, Srirojn B, Latkin CA, Aramratanna A, Celentano DD: **Evaluation of a peer network intervention trial among young methamphetamine users in Chiang Mai, Thailand**. *SOC SCI MED* 2009, **68**(1):69-79.

33. Weeks MR, Li J, Dickson-Gomez J, Convey M, Martinez M, Radda K, Clair S: **Outcomes of a Peer HIV Prevention Program with Injection Drug and Crack Users: The Risk Avoidance Partnership**. *SUBST USE MISUSE* 2009, **44**(2):253-281.

34. Purcell DW, Latka MH, Metsch LR, Latkin CA, Gómez CA, Mizuno Y, Arnsten JH, Wilkinson JD, Knight KR, Knowlton AR *et al*: **Results From a Randomized Controlled Trial of a Peer-Mentoring Intervention to Reduce HIV Transmission and Increase Access to Care and Adherence to HIV Medications Among HIV-Seropositive Injection Drug Users**. *JAIDS Journal of Acquired Immune Deficiency Syndromes* 2007, **46 Suppl 2, HIV Prevention and Clinical Care for HIV-Positive Injection Drug Users: Lessons from the INSPIRE Study**(Supplement 2

):S35-S47.

35. Garfein RS, Golub ET, Greenberg AE, Hagan H, Hanson DL, Hudson SM, Kapadia F, Latka MH, Ouellet LJ, Purcell DW *et al*: **A peer-education intervention to reduce injection risk behaviors for HIV and hepatitis C virus infection in young injection drug users**. *AIDS (London, England)* 2007, **21**(14):1923-1932.

36. Des Jarlais DC, Kling R, Hammett TM, Ngu D, Liu W, Chen Y, Binh KT, Friedmann P: **Reducing HIV infection among new injecting drug users in the China-Vietnam Cross Border Project**. *AIDS* 2007, **21 Suppl 8**:S109-S114.

37. Broadhead RS, Volkanevsky VL, Rydanova T, Ryabkova M, Borch C, van Hulst Y, Fullerton A, Sergeyev B, Heckathorn DD: **Peer-driven HIV interventions for drug injectors in Russia: First year impact results of a field experiment**. *INT J DRUG POLICY* 2006, **17**(5):379-392.

38. Hammett TM: **Community Attitudes Toward HIV Prevention for Injection Drug Users: Findings from a Cross-Border Project in Southern China and Northern Vietnam**. *Journal of Urban Health: Bulletin of the New York Academy of Medicine* 2005, **82**(3_suppl_4):v34-v42.

39. Latkin CA, Sherman S, Knowlton A: **HIV prevention among drug users: Outcome of a network-oriented peer outreach intervention.** *HEALTH PSYCHOL* 2003, **22**(4):332-339.

40. Booth RE, Davis JM, Dvoryak S, Brewster JT, Lisovska O, Strathdee SA, Latkin CA: **HIV incidence among people who inject drugs (PWIDs) in Ukraine: results from a clustered randomised trial**. *The Lancet HIV* 2016, **3**(10):e482-e489.

41. Isac S, Ramesh BM, Rajaram S, Washington R, Bradley JE, Reza-Paul S, Beattie TS, Alary M, Blanchard JF, Moses S: **Changes in HIV and syphilis prevalence among female sex workers from three serial cross-sectional surveys in Karnataka state, South India**. *BMJ OPEN* 2015, **5**(3):e7106.

42. Traore IT, Meda N, Hema NM, Ouedraogo D, Some F, Some R, Niessougou J, Sanon A, Konate I, Van De Perre P *et al*: **HIV prevention and care services for female sex workers: efficacy of a targeted community-based intervention in Burkina Faso**. *J INT AIDS SOC* 2015, **18**(1):20088.

43. Surratt HL, O Grady C, Kurtz SP, Levi-Minzi MA, Chen M: **Outcomes of a Behavioral Intervention to Reduce HIV Risk Among Drug-involved Female Sex Workers**. *AIDS BEHAV* 2014, **18**(4):726-739.

44. Kang D, Tao X, Liao M, Li J, Zhang N, Zhu X, Sun X, Lin B, Su S, Hao L *et al*: **An integrated individual, community, and structural intervention to reduce HIV/STI risks among female sex workers in China**. *BMC PUBLIC HEALTH* 2013, **13**(1

):717.

45. Ang A, Morisky DE: **A Multilevel Analysis of the Impact of Socio-Structural and Environmental Influences on Condom Use Among Female Sex Workers**. *AIDS BEHAV* 2012, **16**(4):934-942.

46. Xiushi Yang GXXL: **The efficacy of a peer-assisted multi-component behavioral intervention among female entertainment workers in China: an initial assessment**. *AIDS CARE* 2011, **11**(23):1509-1518.

47. Thilakavathi S, Boopathi K, Girish Kumar CP, Santhakumar A, Senthilkumar R, Eswaramurthy C, Ilaya Bharathy V, Ramakrishnan L, Thongamba G, Adhikary R *et al*: **Assessment of the scale, coverage and outcomes of the Avahan HIV prevention program for female sex workers in Tamil Nadu, India: is there evidence of an effect?** *BMC PUBLIC HEALTH* 2011, **11 Suppl 6**(Suppl 6

):S3.

48. Rachakulla HK, Kodavalla V, Rajkumar H, Prasad SPV, Kallam S, Goswami P, Dale J, Adhikary R, Paranjape R, Brahmam GNV: **Condom use and prevalence of syphilis and HIV among female sex workers in Andhra Pradesh, India - following a large-scale HIV prevention intervention**. *BMC PUBLIC HEALTH* 2011, **11 Suppl 6**(Suppl 6

):S1.

49. Konate I, Traore L, Ouedraogo A, Sanon A, Diallo R, Ouedraogo JL, Huet C, Millogo I, Andonaba JB, Mayaud P *et al*: **Linking HIV prevention and care for community interventions among high-risk women in Burkina Faso--the ARNS 1222 "Yerelon" cohort**. *J Acquir Immune Defic Syndr* 2011, **57 Suppl 1**:S50-S54.

50. Davey-Rothwell MA, Tobin K, Yang C, Sun CJ, Latkin CA: **Results of a Randomized Controlled Trial of a Peer Mentor HIV/STI Prevention Intervention for Women Over an 18 Month Follow-Up**. *AIDS BEHAV* 2011, **15**(8):1654-1663.

51. Mainkar MM, Pardeshi DB, Dale J, Deshpande S, Khazi S, Gautam A, Goswami P, Adhikary R, Ramanathan S, George B *et al*: **Targeted interventions of the Avahan program and their association with intermediate outcomes among female sex workers in Maharashtra, India**. *BMC PUBLIC HEALTH* 2011, **11 Suppl 6**:S2.

52. Ramesh BM, Beattie TSH, Shajy I, Washington R, Jagannathan L, Reza-Paul S, Blanchard JF, Moses S: **Changes in risk behaviours and prevalence of sexually transmitted infections following HIV preventive interventions among female sex workers in five districts in Karnataka state, south India**. *SEX TRANSM INFECT* 2010, **86**(Suppl 1):i17-i24.

53. Luchters S, Chersich MF, Rinyiru A, Barasa MS, King'Ola N, Mandaliya K, Bosire W, Wambugu S, Mwarogo P, Temmerman M: **Impact of five years of peer-mediated interventions on sexual behavior and sexually transmitted infections among female sex workers in Mombasa, Kenya**. *BMC PUBLIC HEALTH* 2008, **8**:143.

54. Xue H, Luo Z, Zhu Z, Yang X, Yang L, Yang J, Duo L, Liu W: **[Intervention caused changes in high risk sex behaviors among female sex workers from Vietnam in Yunnan, 2009-2013]**. *Zhonghua Liu Xing Bing Xue Za Zhi* 2015, **36**(9):941-944.

55. Geibel S, King'Ola N, Temmerman M, Luchters S: **The impact of peer outreach on HIV knowledge and prevention behaviours of male sex workers in Mombasa, Kenya**. *SEX TRANSM INFECT* 2012, **88**(5):357-362.

56. Rhodes SD, McCoy TP, Vissman AT, DiClemente RJ, Duck S, Hergenrather KC, Foley KL, Alonzo J, Bloom FR, Eng E: **A Randomized Controlled Trial of a Culturally Congruent Intervention to Increase Condom Use and HIV Testing Among Heterosexually Active Immigrant Latino Men**. *AIDS BEHAV* 2011, **15**(8):1764-1775.

57. Hoke TH, Feldblum PJ, Damme KV, Nasution MD, Grey TW, Wong EL, Ralimamonjy L, Raharimalala L, Rasamindrakotroka A: **Randomised controlled trial of alternative male and female condom promotion strategies targeting sex workers in Madagascar**. *SEX TRANSM INFECT* 2007, **83**(6):448-453.

58. Ishika Basu SJMJ: **HIV Prevention Among Sex Workers in India**. *Journal of acquired immune deficiency syndrome* 2004, **3**(36):845-852.

59. Shaikh S, Mburu G, Arumugam V, Mattipalli N, Aher A, Mehta S, Robertson J: **Empowering communities and strengthening systems to improve transgender health: outcomes from the Pehchan programme in India**. *J INT AIDS SOC* 2016, **19**(3 (Suppl 2)).

60. Pawa D, Firestone R, Ratchasi S, Dowling O, Jittakoat Y, Duke A, Mundy G: **Reducing HIV risk among transgender women in Thailand: a quasi-experimental evaluation of the sisters program**. *PLOS ONE* 2013, **8**(10):e77113.
