# Supplementary material for: Peer education for HIV prevention among high-risk groups: a systematic review and meta-analysis
Source: BMC Infect Dis. 2020 May 12;20:338. doi: 10.1186/s12879-020-05003-9 (PMC7218508; doi:10.1186/s12879-020-05003-9)
Supplement: Supplementary file 2 — Additional file 2: Table S1. Study quality assessment. Table S2. The results of egger test. [file 12879_2020_5003_MOESM2_ESM.pdf]

Supplemental table 1 Study quality assessment

Study	Cohort	Control	Pre/post intervention	Random assignment of participants	Follow-up rate of 80% or more	Comparison groups equivalent on socio-demographics	Comparison groups equivalent on outcome measure	Sample size>100	Final score out of 8
AL Wirtz 2015	1	0	1	0	1	0	0	1	4
B M Ramesh 2009	0	0	1	0	0	0	0	1	2
Bindya Jain 2014	1	0	1	0	1	0	0	1	4
Carl A. Latkin 2003	1	1	1	1	1	1	1	1	8
David W. Purcell 2007	1	1	1	1	1	1	1	1	8
Dianmin Kang 2013	0	0	0	0	0	0	0	1	1
Don C.Des Jarlais 2007	0	0	1	0	0	0	0	1	2
Duangta Pawa 2013	1	1	1	0	1	1	1	1	7
Guodong Mi 2015	1	1	0	1	1	1	1	1	7
Hari Kumar Rachakulla 2011	0	0	1	0	0	0	0	1	2
Hilary L.Surratt 2014	1	1	1	1	1	1	1	1	8
Hongbo Zhang 2010	1	0	1	0	0	0	0	1	3
Hongjing Yan 2014	1	0	1	0	1	0	0	1	4
Irving F. Hoffman 2013	1	1	1	1	0	1	1	1	7
Ishika Basu 2004	1	1	1	0	1	1	1	1	7
Isidore T Traore 2015	1	0	1	0	1	0	0	1	4
Issouf Konate 2011	1	0	1	0	1	0	0	1	4
Joseph T.F.Lau 2012	1	1	1	1	1	1	1	0	7
Karin Elizabeth Tobin 2011	1	1	1	1	1	1	1	1	8

Supplemental table 1 (continued) Study quality assessment

Study	Cohort	Control	Pre/post intervention	Random assignment of participants	Follow-up rate of 80% or more	Comparison groups equivalent on socio-demographics	Comparison groups equivalent on outcome measure	Sample size>100	Final score out of 8
M.Yun Gao 2007	1	1	1	0	1	1	1	1	7
Mandar M Mainkar 2011	0	0	1	0	0	0	0	1	2
Marco A.Hidalgo 2015	1	1	1	0	1	1	1	1	7
Margaret R.Weeks 2009	1	0	1	0	0	0	0	1	3
Nai-Ying Ko 2013	1	1	1	0	1	0	1	1	6
Robert E. Booth 2011	1	1	1	0	1	0	1	1	6
Robert E. Booth 2016	1	1	1	1	1	1	1	1	8
Robert S. Broadhead 2006	1	1	1	0	0	1	1	1	6
S Thilakavathi 2011	0	0	1	0	0	0	0	1	2
Scott D. Rhodes 2011	1	1	1	1	1	1	1	1	8
Scott Geibel 2012	0	0	1	0	0	0	0	1	2
Sean D Young 2013	1	1	1	1	1	1	1	1	8
Sean D Young 2015	1	1	1	1	1	1	1	1	8
Shajt Isac 2015	0	0	1	0	0	0	0	1	2
Simran Shaikh 2016	0	0	1	0	0	0	0	1	2
Song-Ying Shen 2011	1	1	1	0	0	1	1	1	6
Stanley Luchters 2008	0	0	1	0	0	0	0	1	2
Susan G.Sherman 2009	1	1	1	1	1	1	1	1	8
Sylvia Adebajo 2015	0	0	1	0	0	0	0	1	2
Theodore M.Hammett 2005	0	0	1	0	0	0	0	1	2
Katherine P.Theall 2015	0	0	1	0	0	0	0	1	2

Supplemental table 1 (continued) Study quality assessment

Study	Cohort	Control	Pre/post intervention	Random assignment of participants	Follow-up rate of 80% or more	Comparison groups equivalent on socio-demographics	Comparison groups equivalent on outcome measure	Sample size>100	Final score out of 8
Theodore M.Hammett 2011	0	0	1	0	0	0	0	1	2
Theodore M.Hammett 2012	0	1	1	0	0	1	1	1	5
Thilakavathi S.2013	0	0	1	0	0	0	0	1	2
Vivian F.Go 2013	1	1	1	1	1	1	1	1	8
Xiushi Yang 2011	1	1	1	0	0	1	1	1	6
Yu Liu 2018	1	1	1	1	1	1	1	1	8
Yuri A.Amirkhanian 2005	1	1	1	1	1	1	1	1	8
Yuwen Duan 2013	1	1	1	0	0	1	1	1	6
Zhujunli 2008	1	0	1	0	0	0	0	1	3
Sylvia Adebajo 2015	0	0	1	0	0	0	0	1	2
Aleksandra Mihailovic 2015	1	1	1	1	1	1	1	1	8
Carl Latkin 2013	1	1	1	1	0	1	1	1	7
Mary E.Mackesy-Amiti 2013	1	1	1	1	1	1	1	1	8
Richard S. Garfein 2007	1	1	0	1	0	1	1	1	6
Carl latkin 2009	1	1	1	1	1	1	1	1	8
M.A Davey-Rothwell 2011	1	1	0	1	1	1	1	1	7
Theresa H Hoke 2007	1	1	0	1	1	0	1	1	6
Lisa M Williamson 2001	0	0	1	0	0	0	0	1	2
Prabuddhagopal Goswami 2012	0	0	1	0	0	0	0	1	2

Supplemental table 2 the results of egger test

	t	*P*
unprotected sex in high risk groups	-2.6445	0.0267*
HIV testing	2.2945	0.03774*
HIV prevalence	-0.9702	0.3604
Equipment sharing	0.1692	0.8681
General condom use	1.2654	0.2152
Consistent condom use	1.3045	0.2085
Condom use with regular partners	0.6354	0.5341
Condom use with casual partners	1.0015	0.3349

*P<0.05

Supplemental table 3 Results of meta regression.

	HIV testing		Equipment sharing		Condom use	
	estimate	P	Estimate	P	Estimate	P
Time	0.0416	0.0345*	-0.3665	0.938***	0.0461	0.574
East Asia	0.3235	0.6206	-0.0469	0.9334	Null	Null
Europe	-0.5752	0.5576	0.3039	0.5964	Null	Null
Africa	Null	Null	Null	Null	0.2639	0.4379
Central Asia	Null	Null	Null	Null	0.1890	0.5675
IDUs	-0.258	0.7761	Null	Null	0.198	0.7643
MSM	0.644	0.2861	Null	Null	-0.0068	0.9896
FSWs	Null	Null	Null	Null	0.4108	0.4048

Significant codes: 0 ‘***’ 0.001 ‘**’ 0.01 ‘*’ 0.05 ‘.’ 0.1 ‘ ’ 1
