# Supplementary material for: Peer education for HIV prevention among high-risk groups: a systematic review and meta-analysis
Source: BMC Infect Dis. 2020 May 12;20:338. doi: 10.1186/s12879-020-05003-9 (PMC7218508; doi:10.1186/s12879-020-05003-9)
Supplement: Supplementary file 3 — Additional file 3: Figure S1. The sensitivity analysis of the unprotected sex. Figure S2. The sensitivity analysis of the HIV testing. Figure S3. The sensitivity analysis of the equipment use. Figure S The sensitivity analysis of the HIV prevalence. Figure S5. The sensitivity analysis of the general condom use. Figure S6. The sensitivity analysis of the consistent condom use. Figure S7. The sensitivity analysis of the condom use with casual partners. Figure S8. The sensitivity analysis of the condom use with regular partners. [file 12879_2020_5003_MOESM3_ESM.pdf]

Study	TE	seTE	Odds Ratio	OR	95%−CI	Weight (fixed)	Weight (random)
Scott D.Rhodes 2011	1.26	0.5128		3.52	[1.29; 9.62]	0.8%	3.9%
Dianmin Kang 2013	0.53	0.1455		1.70	[1.28; 2.26]	10.1%	7.0%
S Thilakavachi 2011	1.46	0.1642		4.30	[3.12; 5.93]	7.9%	6.9%
Shajy Isac 2015	1.59	0.1034		4.90	[4.00; 6.00]	20.0%	7.3%
B M Ramesh 2010	0.68	0.1150		1.98	[1.58; 2.48]	16.2%	7.2%
Hari Kumar Rachakulla 2011	1.09	0.1345		2.98	[2.29; 3.88]	11.8%	7.1%
Isidore T Traore 2015	0.78	0.2789		2.18	[1.26; 3.77]	2.7%	5.9%
Stanley Luchters 2008	1.28	0.2720		3.60	[2.11; 6.14]	2.9%	6.0%
Mandar M Mainkar 2011	1.51	0.2150		4.53	[2.97; 6.90]	4.6%	6.5%
Xue Haoming 2015	1.82	0.7540		6.19	[1.41; 27.13]	0.4%	2.5%
Xiushi Yang 2011	0.29	0.1863		1.34	[0.93; 1.93]	6.2%	6.7%
Simran Shaikh 2016	2.03	0.3046		7.63	[4.20; 13.86]	2.3%	5.7%
Duangta Oawa 2013	1.32	0.4990		3.75	[1.41; 9.97]	0.9%	4.0%
Issouf Konate 2011	2.43	0.2286		11.41	[7.29; 17.86]	4.1%	6.4%
Scott Geibel 2012	0.57	0.3529		1.76	[0.88; 3.51]	1.7%	5.2%
Theresa H Hoke 2007	0.81	0.1969		2.25	[1.53; 3.31]	5.5%	6.6%
Ishika Basu 2004	0.77	0.3389		2.16	[1.11; 4.20]	1.9%	5.3%
**Fixed effect model**				**3.06**	**[2.79; 3.34]**	**100.0%**	**--**
**Random effects model**				**3.19**	**[2.41; 4.23]**	**--**	**100.0%**

Heterogeneity: I^2 = 88%, τ^2 = 0.2734, p < 0.01

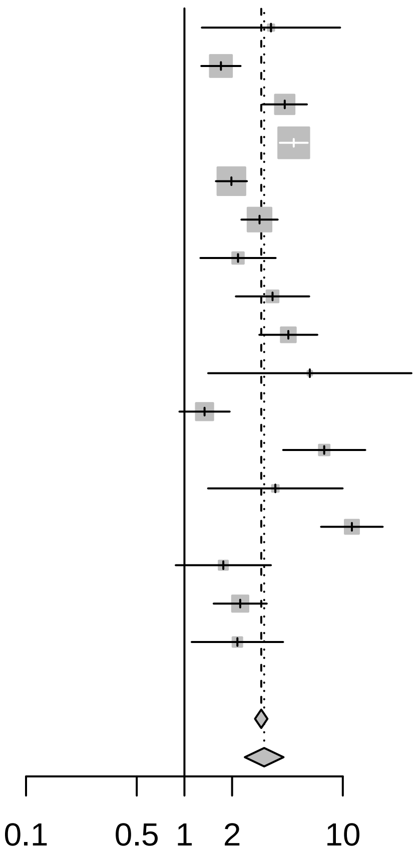

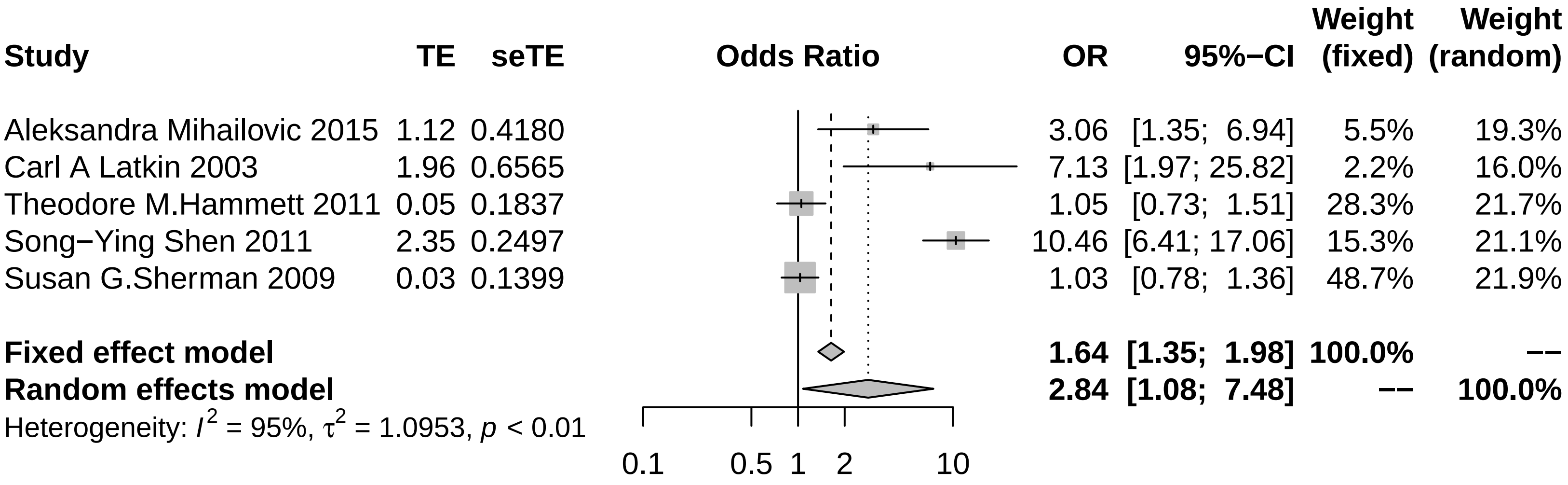
Study
TE
seTE
Odds Ratio
OR
95%−CI
Weight (fixed)
Weight (random)
Aleksandra Mihailovic 2015 1.12 0.4180 3.06 [1.35; 6.94] 5.5% 19.3%
Carl A Latkin 2003 1.96 0.6565 7.13 [1.97; 25.82] 2.2% 16.0%
Theodore M.Hammett 2011 0.05 0.1837 1.05 [0.73; 1.51] 28.3% 21.7%
Song−Ying Shen 2011 2.35 0.2497 10.46 [6.41; 17.06] 15.3% 21.1%
Susan G.Sherman 2009 0.03 0.1399 1.03 [0.78; 1.36] 48.7% 21.9%
Fixed effect model 1.64 [1.35; 1.98] 100.0% −−
Random effects model 2.84 [1.08; 7.48] −− 100.0%
Heterogeneity: I^2 = 95%, τ^2 = 1.0953, p < 0.01
0.1
0.5
1
2
10

Study	TE	seTE	Odds Ratio	OR	95%−CI	Weight (fixed)	Weight (random)
Joseph T.F Lau 2013	0.44	0.1468		1.56	[1.17; 2.08]	16.3%	13.7%
M.Yun Gao 2007	2.67	0.6539		14.37	[3.99; 51.77]	0.8%	3.1%
Nai−Ying Ko 2013	0.21	0.1622		1.23	[0.89; 1.69]	13.4%	13.1%
Hongbo Zhang 2010	0.41	0.1202		1.50	[1.19; 1.90]	24.4%	14.6%
Prabuddhagopal Goswami 2012	1.53	0.6087		4.62	[1.40; 15.23]	1.0%	3.5%
Yuwen Duan 2013	1.20	0.2975		3.33	[1.86; 5.97]	4.0%	8.7%
AL Wietz 2015	0.70	0.3473		2.01	[1.02; 3.97]	2.9%	7.4%
Thilakavashi Subramanian 2013	0.64	0.1865		1.90	[1.32; 2.74]	10.1%	12.3%
Guodong Mi 2015	−0.27	0.2803		0.76	[0.44; 1.32]	4.5%	9.2%
Zhu Junli 2008	0.51	0.1248		1.66	[1.30; 2.12]	22.6%	14.4%
**Fixed effect model**				**1.60**	**[1.43; 1.80]**	**100.0%**	**−−**
**Random effects model**				**1.76**	**[1.37; 2.26]**	**−−**	**100.0%**

Heterogeneity: $I^2 = 72\%$, $\tau^2 = 0.0965$, $p < 0.01$

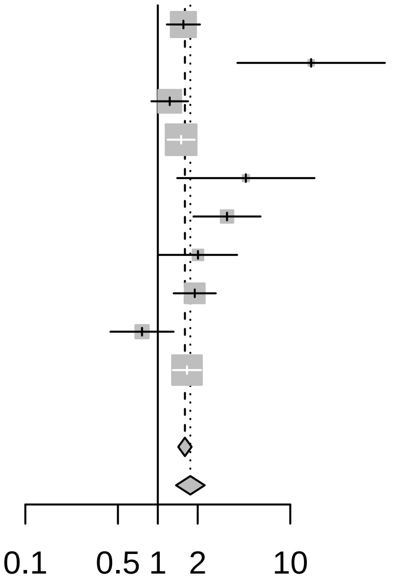

Study	TE	seTE	OR	95%−CI	Weight (fixed)	Weight (random)
Dianmin Kang 2013	1.19	0.1162	3.30	[2.63; 4.14]	17.9%	8.9%
S Thilakavachi 2011	1.70	0.1801	5.50	[3.86; 7.83]	7.4%	8.2%
Shajy Isac 2015	0.99	0.1229	2.70	[2.12; 3.44]	16.0%	8.8%
Hari Kumar Rachakulla 2011	0.79	0.1208	2.20	[1.74; 2.79]	16.5%	8.8%
Isidore T Traore 2015	0.78	0.3246	2.19	[1.16; 4.14]	2.3%	6.2%
Stanley Luchters 2008	1.28	0.2720	3.60	[2.11; 6.14]	3.3%	6.9%
Mandar M Mainkar 2011	1.61	0.2646	4.99	[2.97; 8.38]	3.4%	7.0%
Scott Geibel 2012	0.68	0.2170	1.97	[1.29; 3.01]	5.1%	7.7%
Nai−Ying Ko 2013	0.21	0.1622	1.23	[0.89; 1.69]	9.2%	8.4%
Prabuddhagopal Goswami 2012	1.75	0.8465	5.78	[1.10; 30.37]	0.3%	2.1%
Yuwen Duan 2013	1.33	0.6474	3.78	[1.06; 13.44]	0.6%	3.1%
AL Wietz 2015	0.87	0.4308	2.38	[1.02; 5.54]	1.3%	4.9%
Thilakavashi Subramanian 2013	1.44	0.2415	4.20	[2.62; 6.74]	4.1%	7.3%
Zhu Junli 2008	0.24	0.1412	1.27	[0.96; 1.68]	12.1%	8.6%
Carl A Latkin 2003	1.96	0.6565	7.13	[1.97; 25.82]	0.6%	3.0%
**Fixed effect model**			**2.51**	**[2.28; 2.77]**	**100.0%**	**−−**
**Random effects model**			**2.79**	**[2.13; 3.66]**	**−−**	**100.0%**

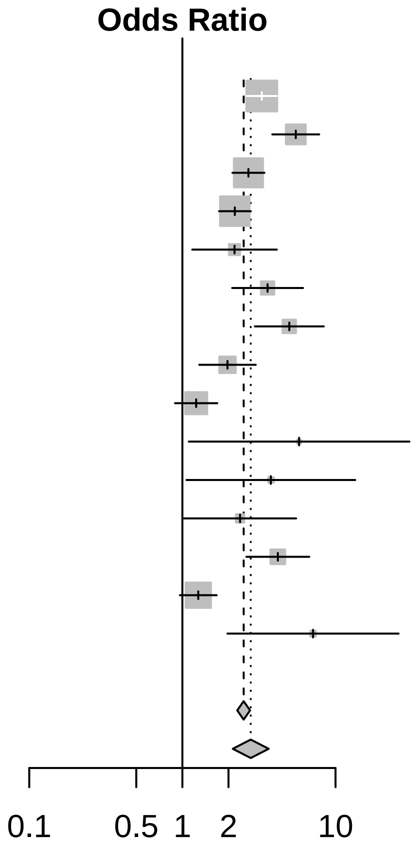

Heterogeneity: $I^2 = 84\%$, $\tau^2 = 0.2002$, $p < 0.01$

Study	TE	seTE	Odds Ratio	OR	95%−CI	Weight (fixed)	Weight (random)
Dianmin Kang 2013	0.53	0.1455		1.70	[1.28; 2.26]	9.3%	6.2%
S Thilakavachi 2011	1.46	0.1642		4.30	[3.12; 5.93]	7.3%	6.1%
Shajy Isac 2015	1.59	0.1034		4.90	[4.00; 6.00]	18.4%	6.3%
Hari Kumar Rachakulla 2011	0.10	0.2629		1.10	[0.66; 1.84]	2.8%	5.7%
Isidore T Traore 2015	−0.34	0.1285		0.71	[0.55; 0.91]	11.9%	6.2%
Mandar M Mainkar 2011	−0.46	0.1797		0.63	[0.44; 0.90]	6.1%	6.1%
Xue Haoming 2015	1.82	0.7540		6.19	[1.41; 27.13]	0.3%	3.4%
Xiushi Yang 2011	0.29	0.1863		1.34	[0.93; 1.93]	5.7%	6.0%
Simran Shaikh 2016	2.03	0.3046		7.63	[4.20; 13.86]	2.1%	5.6%
Issouf Konate 2011	2.43	0.2286		11.41	[7.29; 17.86]	3.8%	5.9%
Scott Geibel 2012	0.57	0.3529		1.76	[0.88; 3.51]	1.6%	5.3%
M.Yun Gao 2007	2.67	0.6539		14.37	[3.99; 51.77]	0.5%	3.9%
Nai−Ying Ko 2013	0.21	0.1622		1.23	[0.89; 1.69]	7.5%	6.1%
Prabuddhagopal Goswami 2012	1.53	0.6087		4.62	[1.40; 15.23]	0.5%	4.1%
Yuwen Duan 2013	1.20	0.2975		3.33	[1.86; 5.97]	2.2%	5.6%
AL Wietz 2015	0.70	0.3473		2.01	[1.02; 3.97]	1.6%	5.4%
Thilakavashi Subramanian 2013	0.64	0.1865		1.90	[1.32; 2.74]	5.7%	6.0%
Zhu Junli 2008	0.51	0.1248		1.66	[1.30; 2.12]	12.7%	6.2%
**Fixed effect model**				**2.06**	**[1.89; 2.25]**	**100.0%**	**−−**
**Random effects model**				**2.45**	**[1.64; 3.66]**	**−−**	**100.0%**

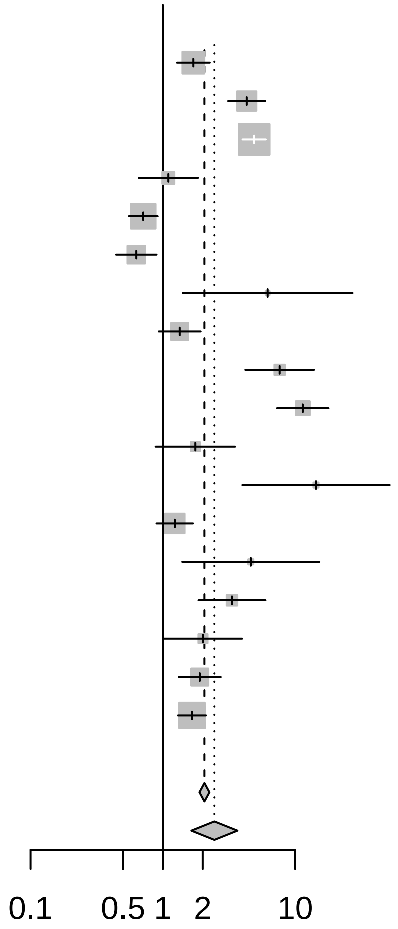

Heterogeneity: $I^2 = 95\%$, $\tau^2 = 0.6579$, $p < 0.01$

Study	TE	seTE	Odds Ratio	OR	95%−CI	Weight (fixed)	Weight (random)
Scott D.Rhodes 2011	1.26	0.5128		3.52	[1.29; 9.62]	0.5%	2.5%
Dianmin Kang 2013	0.53	0.1455		1.70	[1.28; 2.26]	6.5%	5.7%
S Thilakavachi 2011	1.46	0.1642		4.30	[3.12; 5.93]	5.1%	5.6%
Shajy Isac 2015	0.26	0.0956		1.30	[1.08; 1.57]	15.1%	6.1%
Shajy Isac 2015	0.99	0.1229		2.70	[2.12; 3.44]	9.1%	5.9%
Hari Kumar Rachakulla 2011	0.79	0.1208		2.20	[1.74; 2.79]	9.4%	5.9%
Isidore T Traore 2015	0.78	0.3246		2.19	[1.16; 4.14]	1.3%	4.0%
Stanley Luchters 2008	1.28	0.2720		3.60	[2.11; 6.14]	1.9%	4.5%
Mandar M Mainkar 2011	1.61	0.2646		4.99	[2.97; 8.38]	2.0%	4.6%
Xue Haoming 2015	1.82	0.7540		6.19	[1.41; 27.13]	0.2%	1.5%
Xiushi Yang 2011	0.29	0.1863		1.34	[0.93; 1.93]	4.0%	5.4%
Scott Geibel 2012	0.68	0.2170		1.97	[1.29; 3.01]	2.9%	5.1%
Nai−Ying Ko 2013	0.21	0.1622		1.23	[0.89; 1.69]	5.2%	5.6%
Dan Wu 2019	0.29	0.2569		1.33	[0.80; 2.20]	2.1%	4.6%
Thilakavashi Subramanian 2013	0.64	0.1865		1.90	[1.32; 2.74]	4.0%	5.4%
Guodong Mi 2015	−0.27	0.2803		0.76	[0.44; 1.32]	1.8%	4.4%
3Susan G.Sherman 2009	0.22	0.1362		1.24	[0.95; 1.62]	7.4%	5.8%
6Susan G.Sherman 2009	0.20	0.1368		1.22	[0.93; 1.60]	7.4%	5.8%
9Susan G.Sherman 2009	0.20	0.1384		1.22	[0.93; 1.60]	7.2%	5.8%
12Susan G.Sherman 2009	0.03	0.1399		1.03	[0.78; 1.36]	7.0%	5.8%
**Fixed effect model**				**1.66**	**[1.54; 1.78]**	**100.0%**	**−−**
**Random effects model**				**1.80**	**[1.47; 2.21]**	**−−**	**100.0%**

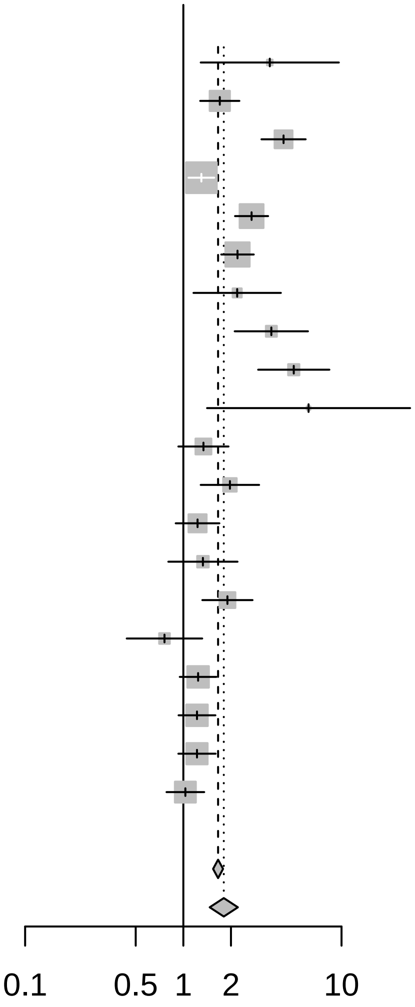

Heterogeneity: $I^2 = 86\%$, $\tau^2 = 0.1702$, $p < 0.01$

The captions

Supplemental figure 9 the forest plot of the condom use among FSWs

Supplemental figure 10 the forest plot of the condom use among IDUs

Supplemental figure 11 the forest plot of the condom use among MSM

Supplemental figure 12 the forest plot of condom use with casual partners

Supplemental figure 13 the forest plot of condom use with regular partners

Supplemental figure 14 the forest plot of consistent condom use
