# Supplementary material for: Peer education for HIV prevention among high-risk groups: a systematic review and meta-analysis
Source: BMC Infect Dis. 2020 May 12;20:338. doi: 10.1186/s12879-020-05003-9 (PMC7218508; doi:10.1186/s12879-020-05003-9)
Supplement: Supplementary file 4 — Additional file 4: Figure S9. The forest plot of the condom use among FSWs. Figure S10. The forest plot of the condom use among IDUs. Figure S11. The forest plot of the condom use among MSM. Figure S12. The forest plot of condom use with casual partners. Figure S13. The forest plot of condom use with regular partners. Figure S14. The forest plot of consistent condom use. [file 12879_2020_5003_MOESM4_ESM.pdf]

The captions

Supplemental figure 1 the sensitivity analysis of the unprotected sex

Supplemental figure 2 the sensitivity analysis of the HIV testing

Supplemental figure 3 the sensitivity analysis of the equipment use

Supplemental figure 4 the sensitivity analysis of the HIV prevalence

Supplemental figure 5 the sensitivity analysis of the general condom use

Supplemental figure 6 the sensitivity analysis of the consistent condom use

Supplemental figure 7 the sensitivity analysis of the condom use with casual partners

Supplemental figure 8 the sensitivity analysis of the condom use with regular partners

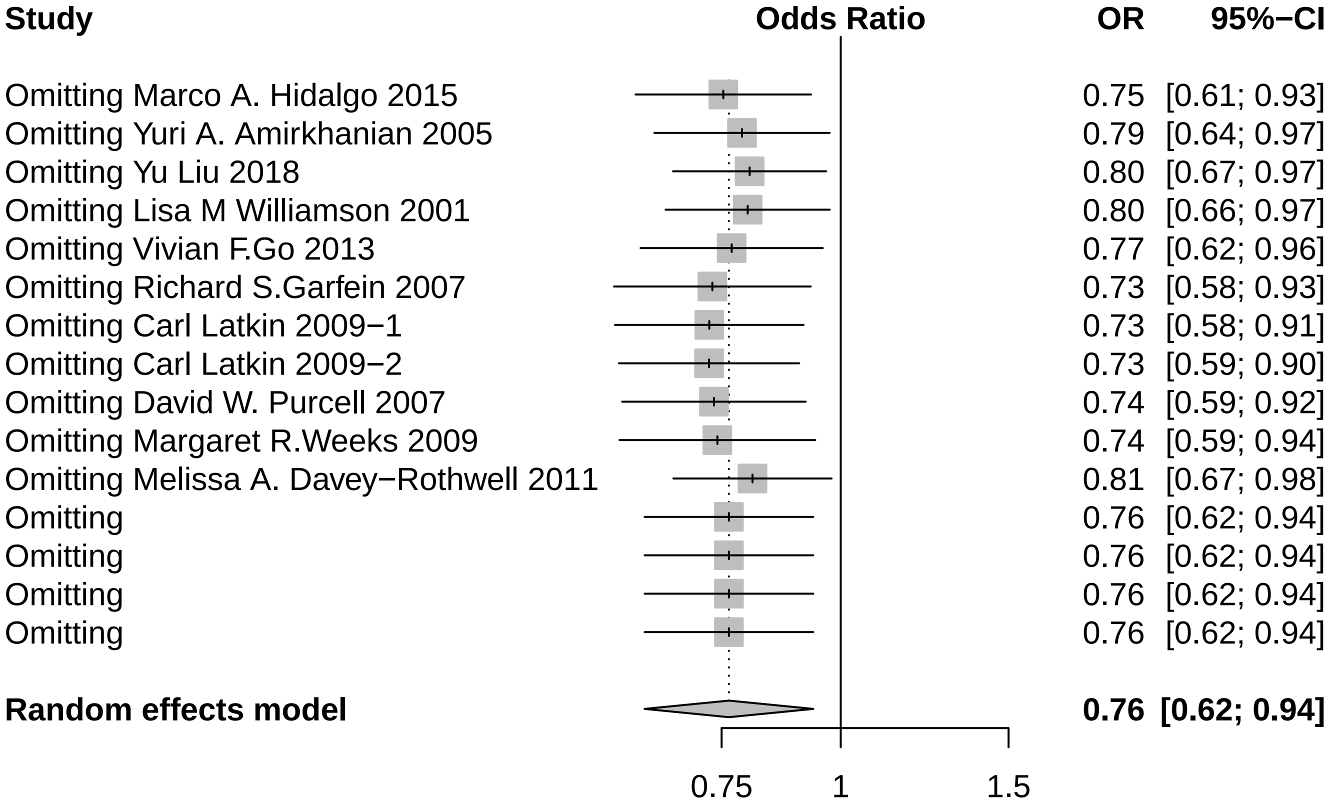
Study
Odds Ratio
OR
95%−CI
Omitting Marco A. Hidalgo 2015 0.75 [0.61; 0.93]
Omitting Yuri A. Amirkhanian 2005 0.79 [0.64; 0.97]
Omitting Yu Liu 2018 0.80 [0.67; 0.97]
Omitting Lisa M Williamson 2001 0.80 [0.66; 0.97]
Omitting Vivian F.Go 2013 0.77 [0.62; 0.96]
Omitting Richard S.Garfein 2007 0.73 [0.58; 0.93]
Omitting Carl Latkin 2009−1 0.73 [0.58; 0.91]
Omitting Carl Latkin 2009−2 0.73 [0.59; 0.90]
Omitting David W. Purcell 2007 0.74 [0.59; 0.92]
Omitting Margaret R.Weeks 2009 0.74 [0.59; 0.94]
Omitting Melissa A. Davey−Rothwell 2011 0.81 [0.67; 0.98]
Omitting 0.76 [0.62; 0.94]
Omitting 0.76 [0.62; 0.94]
Omitting 0.76 [0.62; 0.94]
Omitting 0.76 [0.62; 0.94]
Random effects model 0.76 [0.62; 0.94]
0.75
1
1.5

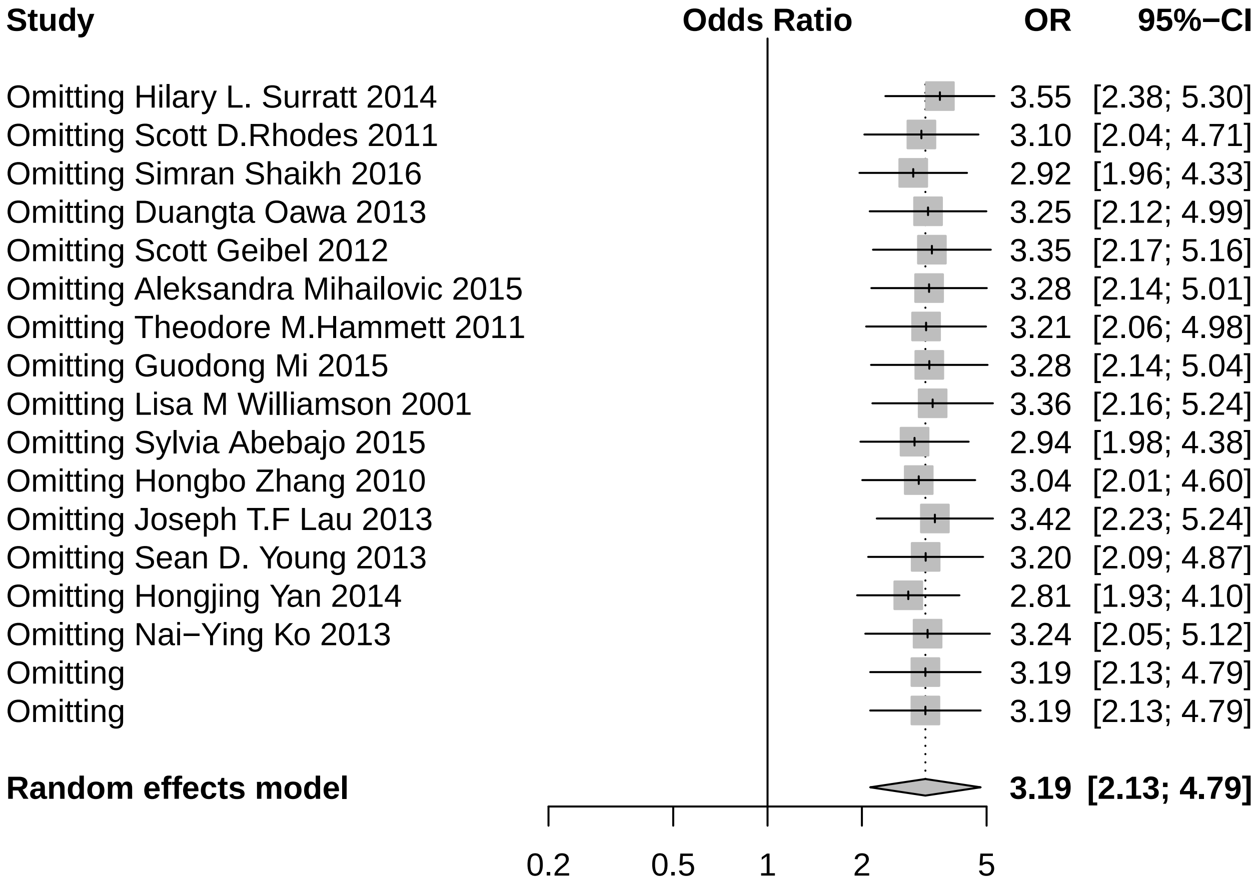
Study
Odds Ratio
OR
95%−CI
Omitting Hilary L. Surratt 2014
3.55 [2.38; 5.30]
Omitting Scott D.Rhodes 2011
3.10 [2.04; 4.71]
Omitting Simran Shaikh 2016
2.92 [1.96; 4.33]
Omitting Duangta Oawa 2013
3.25 [2.12; 4.99]
Omitting Scott Geibel 2012
3.35 [2.17; 5.16]
Omitting Aleksandra Mihailovic 2015
3.28 [2.14; 5.01]
Omitting Theodore M.Hammett 2011
3.21 [2.06; 4.98]
Omitting Guodong Mi 2015
3.28 [2.14; 5.04]
Omitting Lisa M Williamson 2001
3.36 [2.16; 5.24]
Omitting Sylvia Abebajo 2015
2.94 [1.98; 4.38]
Omitting Hongbo Zhang 2010
3.04 [2.01; 4.60]
Omitting Joseph T.F Lau 2013
3.42 [2.23; 5.24]
Omitting Sean D. Young 2013
3.20 [2.09; 4.87]
Omitting Hongjing Yan 2014
2.81 [1.93; 4.10]
Omitting Nai−Ying Ko 2013
3.24 [2.05; 5.12]
Omitting
3.19 [2.13; 4.79]
Omitting
3.19 [2.13; 4.79]
Random effects model
3.19 [2.13; 4.79]
0.2
0.5
1
2
5

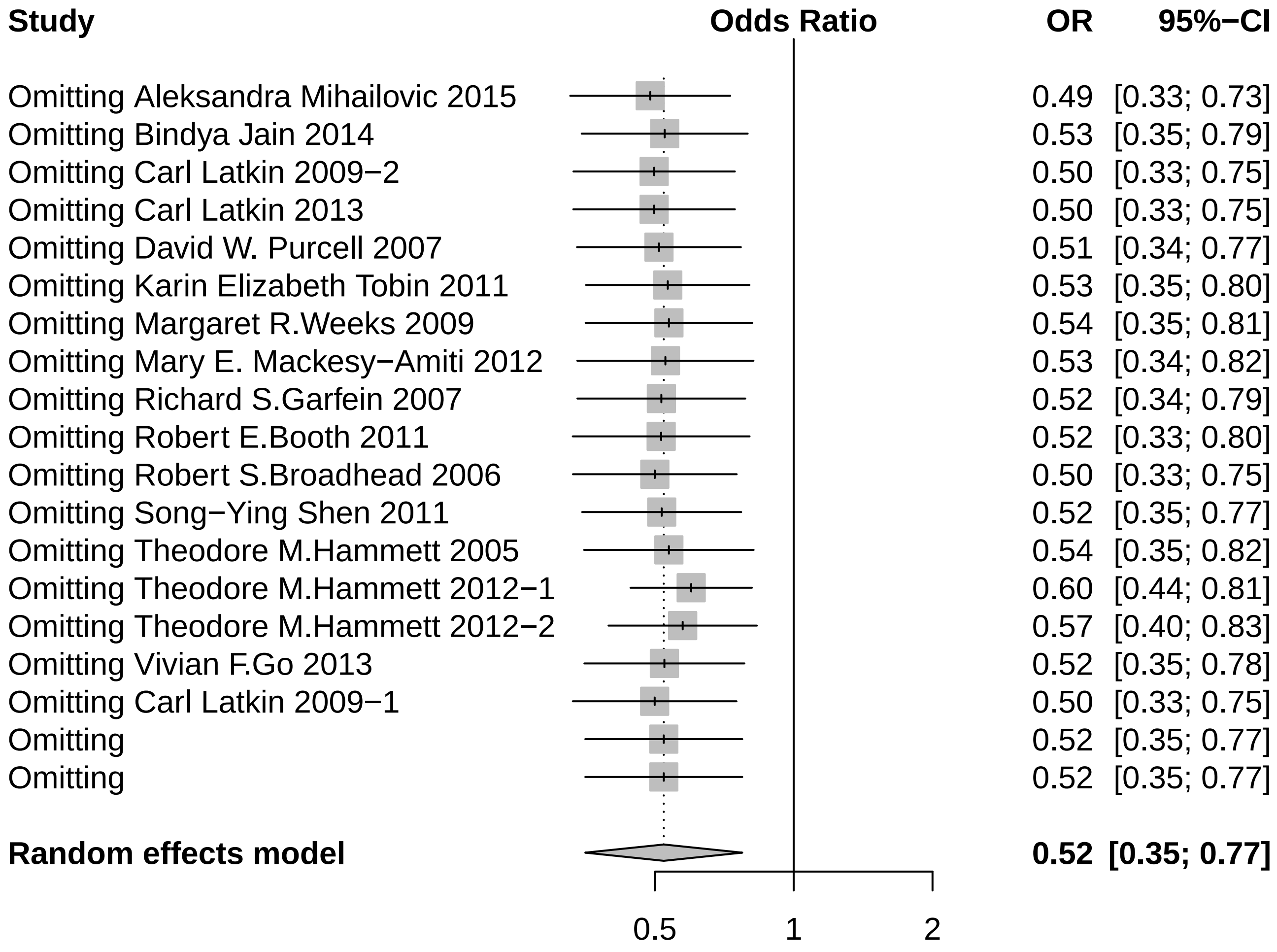
Study
Odds Ratio
OR
95%−CI
Omitting Aleksandra Mihailovic 2015 0.49 [0.33; 0.73]
Omitting Bindya Jain 2014 0.53 [0.35; 0.79]
Omitting Carl Latkin 2009−2 0.50 [0.33; 0.75]
Omitting Carl Latkin 2013 0.50 [0.33; 0.75]
Omitting David W. Purcell 2007 0.51 [0.34; 0.77]
Omitting Karin Elizabeth Tobin 2011 0.53 [0.35; 0.80]
Omitting Margaret R.Weeks 2009 0.54 [0.35; 0.81]
Omitting Mary E. Mackesy−Amiti 2012 0.53 [0.34; 0.82]
Omitting Richard S.Garfein 2007 0.52 [0.34; 0.79]
Omitting Robert E.Booth 2011 0.52 [0.33; 0.80]
Omitting Robert S.Broadhead 2006 0.50 [0.33; 0.75]
Omitting Song−Ying Shen 2011 0.52 [0.35; 0.77]
Omitting Theodore M.Hammett 2005 0.54 [0.35; 0.82]
Omitting Theodore M.Hammett 2012−1 0.60 [0.44; 0.81]
Omitting Theodore M.Hammett 2012−2 0.57 [0.40; 0.83]
Omitting Vivian F.Go 2013 0.52 [0.35; 0.78]
Omitting Carl Latkin 2009−1 0.50 [0.33; 0.75]
Omitting 0.52 [0.35; 0.77]
Omitting 0.52 [0.35; 0.77]
Random effects model 0.52 [0.35; 0.77]
0.5
1
2

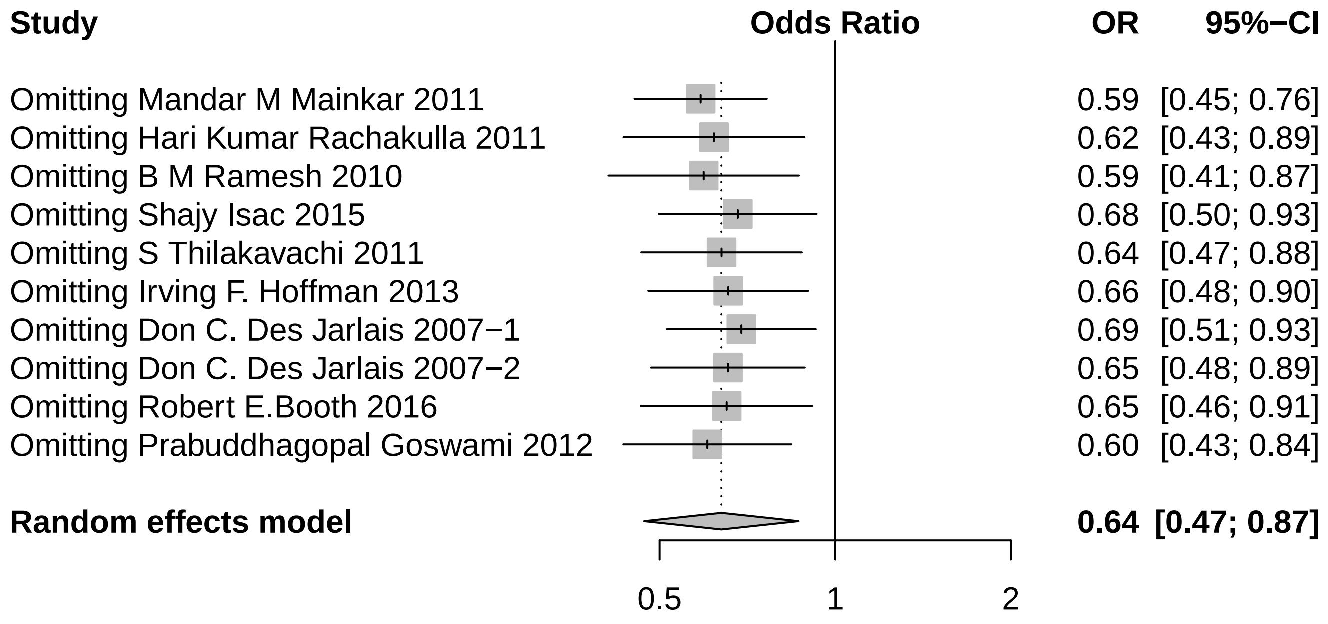
Study
Odds Ratio
OR
95%−CI
Omitting Mandar M Mainkar 2011
0.59 [0.45; 0.76]
Omitting Hari Kumar Rachakulla 2011
0.62 [0.43; 0.89]
Omitting B M Ramesh 2010
0.59 [0.41; 0.87]
Omitting Shajy Isac 2015
0.68 [0.50; 0.93]
Omitting S Thilakavachi 2011
0.64 [0.47; 0.88]
Omitting Irving F. Hoffman 2013
0.66 [0.48; 0.90]
Omitting Don C. Des Jarlais 2007−1
0.69 [0.51; 0.93]
Omitting Don C. Des Jarlais 2007−2
0.65 [0.48; 0.89]
Omitting Robert E.Booth 2016
0.65 [0.46; 0.91]
Omitting Prabuddhagopal Goswami 2012
0.60 [0.43; 0.84]
Random effects model
0.64 [0.47; 0.87]
0.5
1
2

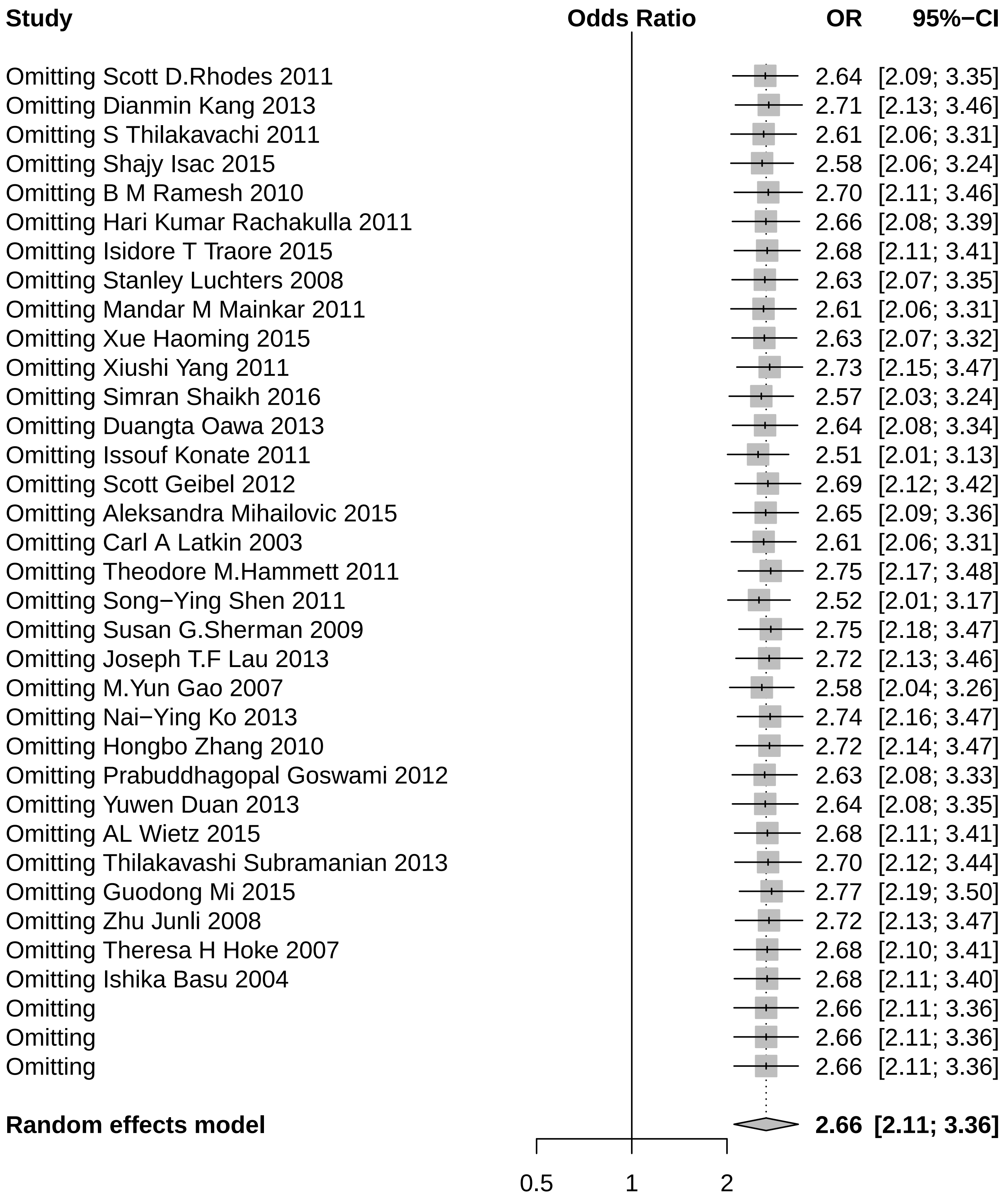

Study
Odds Ratio
OR
95%−CI
Omitting Scott D.Rhodes 2011 2.64 [2.09; 3.35]
Omitting Dianmin Kang 2013 2.71 [2.13; 3.46]
Omitting S Thilakavachi 2011 2.61 [2.06; 3.31]
Omitting Shajy Isac 2015 2.58 [2.06; 3.24]
Omitting B M Ramesh 2010 2.70 [2.11; 3.46]
Omitting Hari Kumar Rachakulla 2011 2.66 [2.08; 3.39]
Omitting Isidore T Traore 2015 2.68 [2.11; 3.41]
Omitting Stanley Luchters 2008 2.63 [2.07; 3.35]
Omitting Mandar M Mainkar 2011 2.61 [2.06; 3.31]
Omitting Xue Haoming 2015 2.63 [2.07; 3.32]
Omitting Xiushi Yang 2011 2.73 [2.15; 3.47]
Omitting Simran Shaikh 2016 2.57 [2.03; 3.24]
Omitting Duangta Oawa 2013 2.64 [2.08; 3.34]
Omitting Issouf Konate 2011 2.51 [2.01; 3.13]
Omitting Scott Geibel 2012 2.69 [2.12; 3.42]
Omitting Aleksandra Mihailovic 2015 2.65 [2.09; 3.36]
Omitting Carl A Latkin 2003 2.61 [2.06; 3.31]
Omitting Theodore M.Hammett 2011 2.75 [2.17; 3.48]
Omitting Song−Ying Shen 2011 2.52 [2.01; 3.17]
Omitting Susan G.Sherman 2009 2.75 [2.18; 3.47]
Omitting Joseph T.F Lau 2013 2.72 [2.13; 3.46]
Omitting M.Yun Gao 2007 2.58 [2.04; 3.26]
Omitting Nai−Ying Ko 2013 2.74 [2.16; 3.47]
Omitting Hongbo Zhang 2010 2.72 [2.14; 3.47]
Omitting Prabuddhagopal Goswami 2012 2.63 [2.08; 3.33]
Omitting Yuwen Duan 2013 2.64 [2.08; 3.35]
Omitting AL Wietz 2015 2.68 [2.11; 3.41]
Omitting Thilakavashi Subramanian 2013 2.70 [2.12; 3.44]
Omitting Guodong Mi 2015 2.77 [2.19; 3.50]
Omitting Zhu Junli 2008 2.72 [2.13; 3.47]
Omitting Theresa H Hoke 2007 2.68 [2.10; 3.41]
Omitting Ishika Basu 2004 2.68 [2.11; 3.40]
Omitting 2.66 [2.11; 3.36]
Omitting 2.66 [2.11; 3.36]
Omitting 2.66 [2.11; 3.36]
Random effects model 2.66 [2.11; 3.36]
0.5
1
2

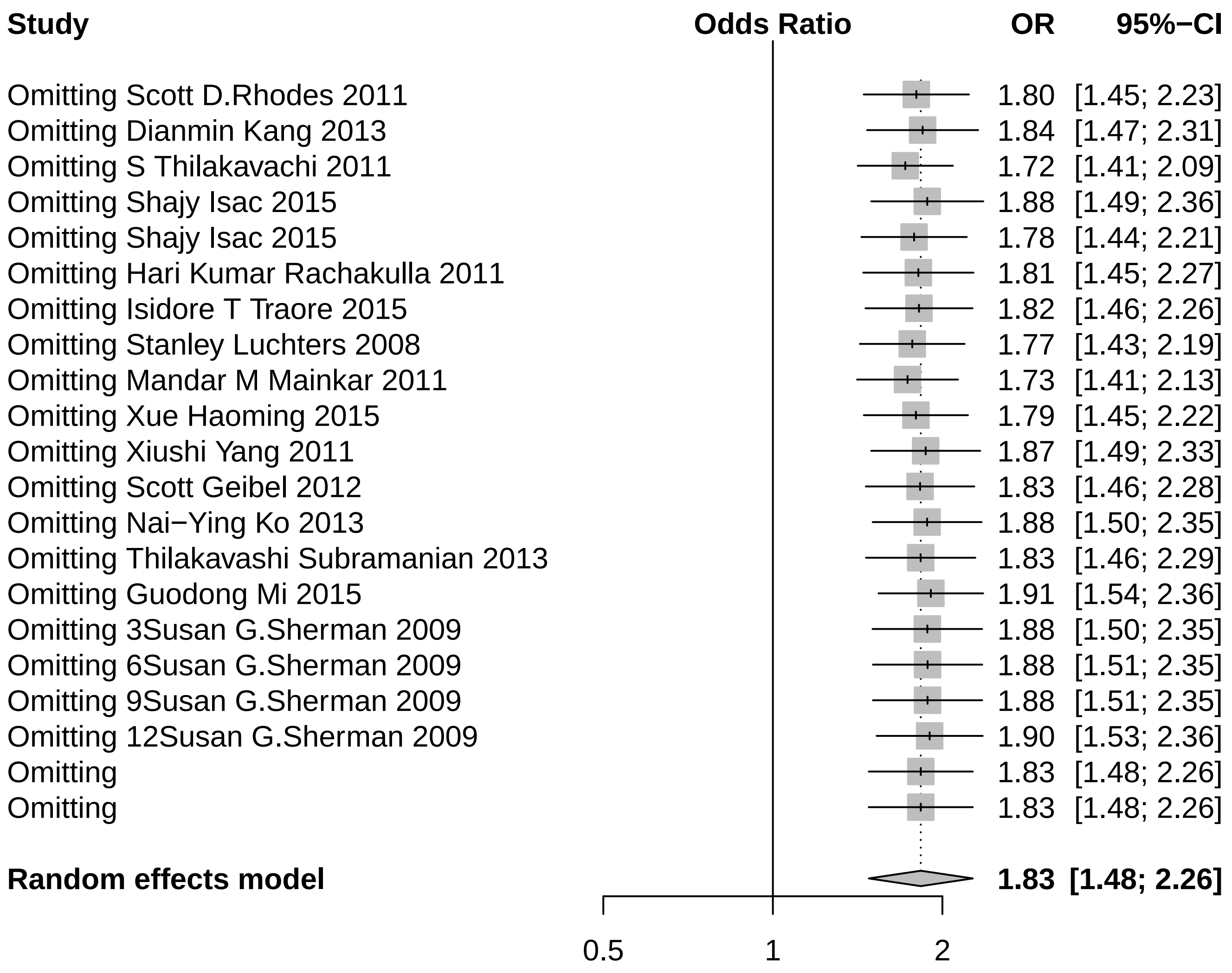
Study
Odds Ratio
OR
95%−CI
Omitting Scott D.Rhodes 2011 1.80 [1.45; 2.23]
Omitting Dianmin Kang 2013 1.84 [1.47; 2.31]
Omitting S Thilakavachi 2011 1.72 [1.41; 2.09]
Omitting Shajy Isac 2015 1.88 [1.49; 2.36]
Omitting Shajy Isac 2015 1.78 [1.44; 2.21]
Omitting Hari Kumar Rachakulla 2011 1.81 [1.45; 2.27]
Omitting Isidore T Traore 2015 1.82 [1.46; 2.26]
Omitting Stanley Luchters 2008 1.77 [1.43; 2.19]
Omitting Mandar M Mainkar 2011 1.73 [1.41; 2.13]
Omitting Xue Haoming 2015 1.79 [1.45; 2.22]
Omitting Xiushi Yang 2011 1.87 [1.49; 2.33]
Omitting Scott Geibel 2012 1.83 [1.46; 2.28]
Omitting Nai−Ying Ko 2013 1.88 [1.50; 2.35]
Omitting Thilakavashi Subramanian 2013 1.83 [1.46; 2.29]
Omitting Guodong Mi 2015 1.91 [1.54; 2.36]
Omitting 3Susan G.Sherman 2009 1.88 [1.50; 2.35]
Omitting 6Susan G.Sherman 2009 1.88 [1.51; 2.35]
Omitting 9Susan G.Sherman 2009 1.88 [1.51; 2.35]
Omitting 12Susan G.Sherman 2009 1.90 [1.53; 2.36]
Omitting 1.83 [1.48; 2.26]
Omitting 1.83 [1.48; 2.26]
Random effects model 1.83 [1.48; 2.26]
0.5
1
2

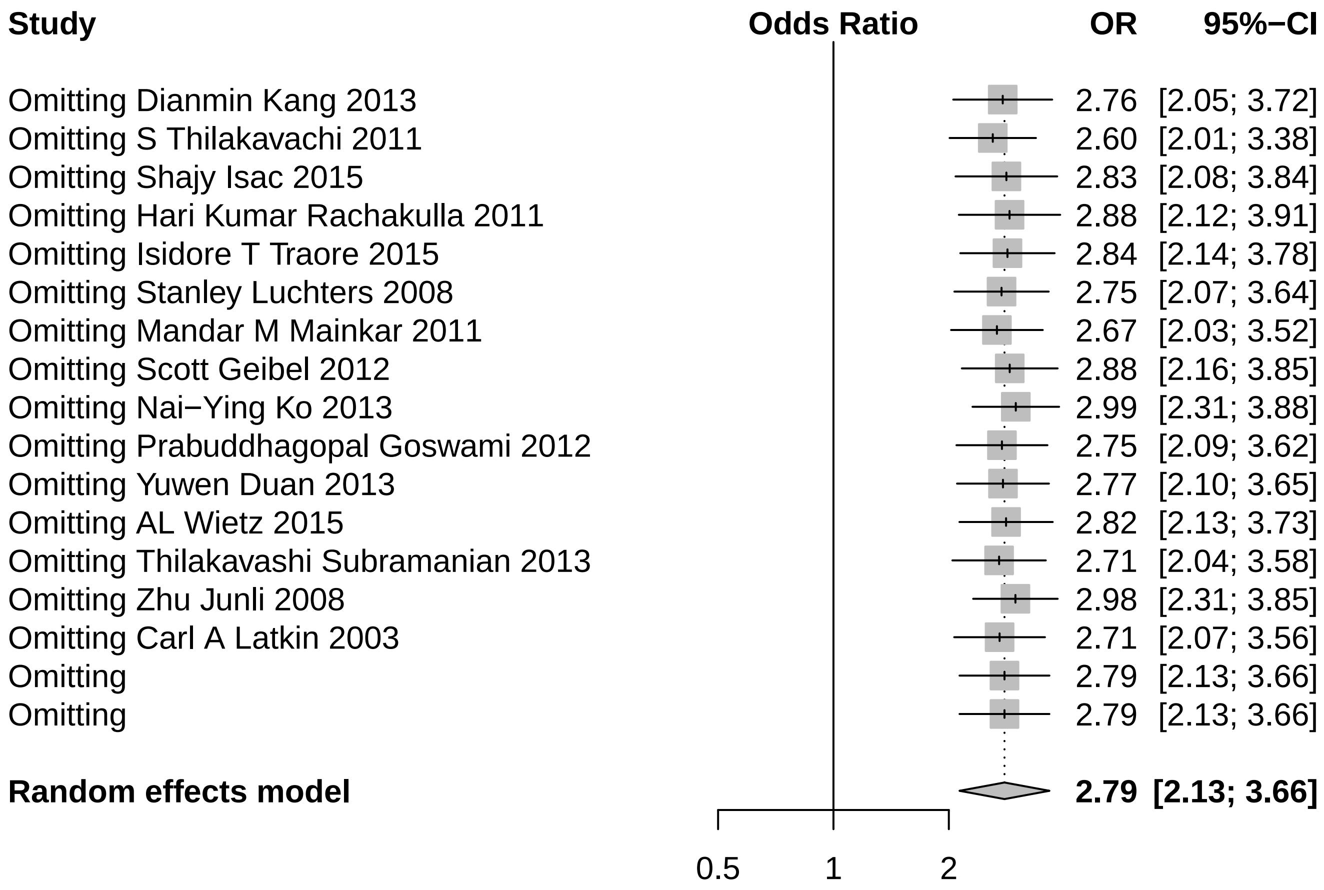
Study
Odds Ratio
OR
95%−CI
Omitting Dianmin Kang 2013 2.76 [2.05; 3.72]
Omitting S Thilakavachi 2011 2.60 [2.01; 3.38]
Omitting Shajy Isac 2015 2.83 [2.08; 3.84]
Omitting Hari Kumar Rachakulla 2011 2.88 [2.12; 3.91]
Omitting Isidore T Traore 2015 2.84 [2.14; 3.78]
Omitting Stanley Luchters 2008 2.75 [2.07; 3.64]
Omitting Mandar M Mainkar 2011 2.67 [2.03; 3.52]
Omitting Scott Geibel 2012 2.88 [2.16; 3.85]
Omitting Nai−Ying Ko 2013 2.99 [2.31; 3.88]
Omitting Prabuddhagopal Goswami 2012 2.75 [2.09; 3.62]
Omitting Yuwen Duan 2013 2.77 [2.10; 3.65]
Omitting AL Wietz 2015 2.82 [2.13; 3.73]
Omitting Thilakavashi Subramanian 2013 2.71 [2.04; 3.58]
Omitting Zhu Junli 2008 2.98 [2.31; 3.85]
Omitting Carl A Latkin 2003 2.71 [2.07; 3.56]
Omitting 2.79 [2.13; 3.66]
Omitting 2.79 [2.13; 3.66]
Random effects model 2.79 [2.13; 3.66]
0.5
1
2

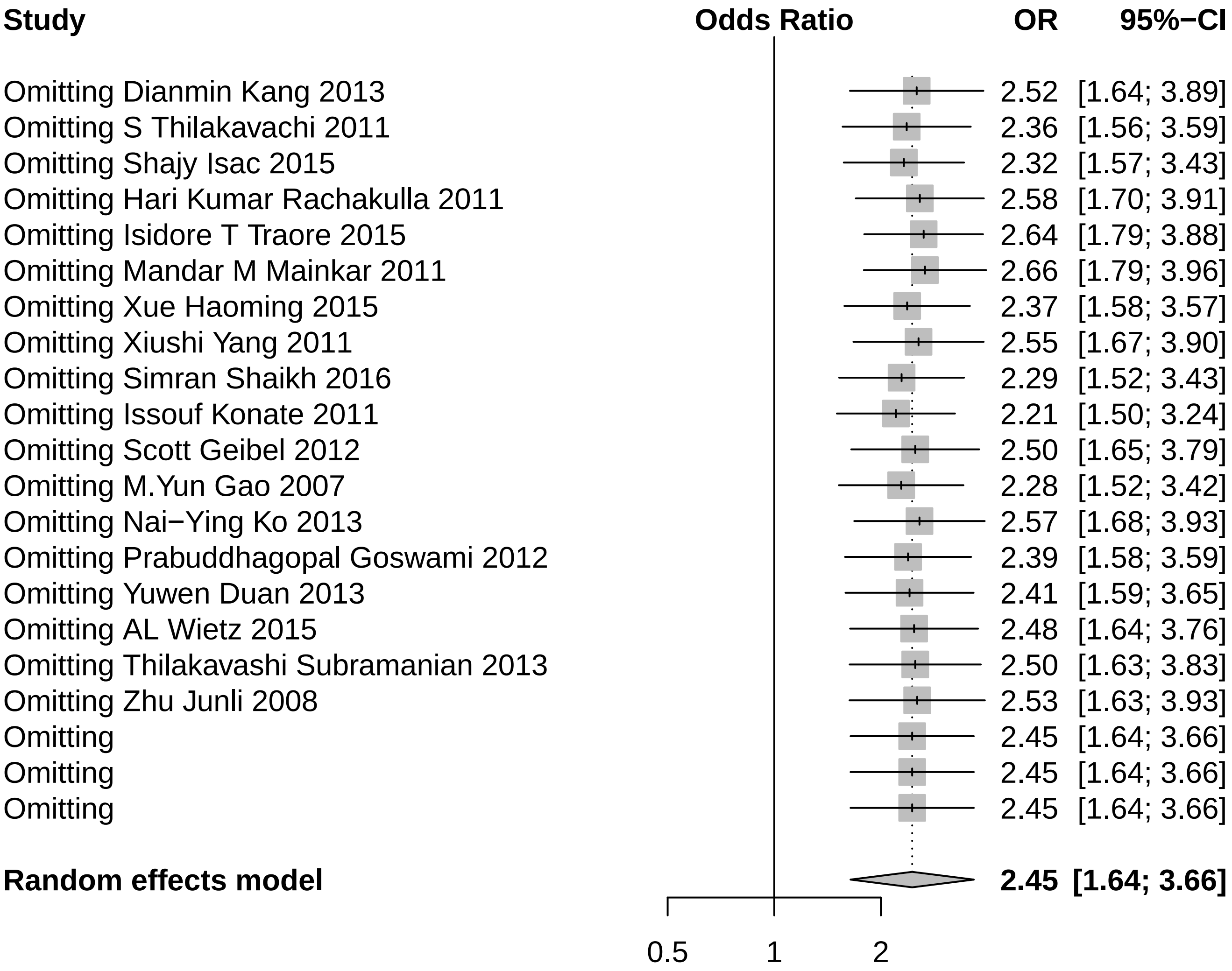

Study
Odds Ratio
OR
95%−CI
Omitting Dianmin Kang 2013 2.52 [1.64; 3.89]
Omitting S Thilakavachi 2011 2.36 [1.56; 3.59]
Omitting Shajy Isac 2015 2.32 [1.57; 3.43]
Omitting Hari Kumar Rachakulla 2011 2.58 [1.70; 3.91]
Omitting Isidore T Traore 2015 2.64 [1.79; 3.88]
Omitting Mandar M Mainkar 2011 2.66 [1.79; 3.96]
Omitting Xue Haoming 2015 2.37 [1.58; 3.57]
Omitting Xiushi Yang 2011 2.55 [1.67; 3.90]
Omitting Simran Shaikh 2016 2.29 [1.52; 3.43]
Omitting Issouf Konate 2011 2.21 [1.50; 3.24]
Omitting Scott Geibel 2012 2.50 [1.65; 3.79]
Omitting M.Yun Gao 2007 2.28 [1.52; 3.42]
Omitting Nai−Ying Ko 2013 2.57 [1.68; 3.93]
Omitting Prabuddhagopal Goswami 2012 2.39 [1.58; 3.59]
Omitting Yuwen Duan 2013 2.41 [1.59; 3.65]
Omitting AL Wietz 2015 2.48 [1.64; 3.76]
Omitting Thilakavashi Subramanian 2013 2.50 [1.63; 3.83]
Omitting Zhu Junli 2008 2.53 [1.63; 3.93]
Omitting 2.45 [1.64; 3.66]
Omitting 2.45 [1.64; 3.66]
Omitting 2.45 [1.64; 3.66]
Random effects model 2.45 [1.64; 3.66]
0.5
1
2
